# *Drosophila* as a Model for Intractable Epilepsy: *Gilgamesh* Suppresses Seizures in *para^bss1^* Heterozygote Flies

**DOI:** 10.1534/g3.113.006130

**Published:** 2013-08-01

**Authors:** Iris C. Howlett, Zeid M. Rusan, Louise Parker, Mark A. Tanouye

**Affiliations:** * Department of Molecular and Cell Biology, Policy and Management, University of California, Berkeley, California 94720; † Department of Environmental Science, Policy and Management, University of California, Berkeley, California 94720

**Keywords:** sodium channel, epilepsy, seizure-suppression, Drosophila

## Abstract

Intractable epilepsies, that is, seizure disorders that do not respond to currently available therapies, are difficult, often tragic, neurological disorders. Na^+^ channelopathies have been implicated in some intractable epilepsies, including Dravet syndrome (Dravet 1978), but little progress has been forthcoming in therapeutics. Here we examine a Drosophila model for intractable epilepsy, the Na^+^ channel gain-of-function mutant *para^bss1^* that resembles Dravet syndrome in some aspects (parker *et al.* 2011a). In particular, we identify second-site mutations that interact with *para^bss1^*, seizure enhancers, and seizure suppressors. We describe one seizure-enhancer mutation named *charlatan (chn)*. The *chn* gene normally encodes an Neuron-Restrictive Silencer Factor/RE1-Silencing Transcription factor transcriptional repressor of neuronal-specific genes. We identify a second-site seizure-suppressor mutation, *gilgamesh (gish)*, that reduces the severity of several seizure-like phenotypes of *para^bss1^/+* heterozygotes. The *gish* gene normally encodes the Drosophila ortholog of casein kinase CK1g3, a member of the CK1 family of serine-threonine kinases. We suggest that CK1g3 is an unexpected but promising new target for seizure therapeutics.

In this study, we examine genetic complexities that underlie seizure-susceptibility by using, as a model, genetic combinations of single-gene mutations in the fruit fly Drosophila: seizure-sensitive, seizure-enhancer, and seizure-suppressor mutations. The study is based on genetic interactions that modify phenotypes in *para^bss1^*, a model for intractable epilepsy (parker *et al.* 2011a). The *para^bss1^* mutant is caused by a gain-of-function mutation in the voltage-gated Na^+^ channel gene that causes extreme seizure sensitivity. In our Drosophila collection, the *para^bss1^* mutant: (1) displays the lowest threshold to evoked seizure-like activity; (2) exhibits the longest paralytic behavior recovery time with prominent episodes of seizure and paralysis that resemble tonic-clonic-like activity; and (3) is the most difficult mutant to suppress by suppressor mutations or antiepileptic drugs (Pavlidis and Tanouye 1995; Kuebler and Tanouye 2000; Kuebler *et al.* 2001; Song and Tanouye 2006).

We describe here the results of a search for new enhancers and suppressors of *para^bss1^*. Because of the potential biomedical usefulness of some of these observations to intractable epilepsies, we are somewhat more deliberate in our descriptions than is usually customary in Drosophila mutant screens. We further describe identification of *charlatan (chn)*, an enhancer of *para^bss1^*, and a *para^bss1^* suppressor named *gilgamesh (gish)*.

## Materials and Methods

### Fly stocks

Drosophila strains were raised on standard cornmeal-molasses agar medium at room temperature (23−25°). The *para* gene is located at map position 1−53.5 and encodes a voltage-gated Na^+^ channel (Loughney *et al.* 1989; Ramaswami and Tanouye, 1989). The bang-sensitive (BS) allele used in this study, *para^bss1^*, previously named *bss^1^*, is the most seizure-sensitive of fly mutants, the most difficult to suppress by mutation and by drug, and is a model for human intractable epilepsy (Ganetzky and Wu 1982; Parker *et al.* 2011a). The *para^bss1^* allele is a gain-of-function mutation caused by a substitution (L1699F) of a highly conserved residue in the third membrane-spanning segment (S3b) of homology domain IV (Parker *et al.* 2011a). In this study, we use *para^bss1^* and *para^bss1^/+* as genetic backgrounds to screen for enhancers and suppressors of seizure, respectively. The *eas* gene is located at 14B on the cytological map and encodes an ethanolamine kinase (Pavlidis *et al.* 1994). The BS allele used in this study is *eas^PC80^*, which is caused by a 2-bp deletion that introduces a frame shift; the resulting truncated protein lacks a kinase domain and abolishes all enzymatic activity (Pavlidis *et al.* 1994). Df(2R)Exel7135=51E2-51E11 contains approximately 22 genes. Df(2R)Exel6056=44A4-44C2 contains approximately 39 genes. Df(2R)Exel6078=58B1-58D1 contains approximately 35 genes. *UAS-gishRNAi* and other *UAS-RNAi* lines were obtained from the Vienna Drosophila RNAi Center. All other lines, including Gal4 drivers and deletion lines, were obtained from the Bloomington Drosophila Stock Center.

### Haplo-deficiency screen for seizure enhancers and suppressors

A screen was designed to detect novel seizure suppressors and enhancers based on haplo-induced changes in *para^bss1^* seizure susceptibility. Using the screen, we examined 200 stocks, each carrying a different Df(2) or Df(3) chromosomal deletion with appropriate CyO, TM3, or TM6 balancer in a *para^bss1^* background. Seizure susceptibility can vary substantially with age, genetic background, and other factors; all comparisons were between age-matched siblings arising from the same cross to minimize variations due to these sources. For Df(2) deletions: female *para^bss1^;+;+* flies were crossed to +/Y;Df(2)/CYO;+ males. Male progeny of the genotype: *para^bss1^/*Y;Df(2)/+;+ were tested for enhancement of BS phenotype compared with their sibling controls (*para^bss1^*/Y;CYO/+;+). Female progeny arising from the same cross, *para^bss1^/+;Df(2)/+*;+, were tested for suppression of the BS phenotype compared with their control siblings (*para^bss1^*/+;CYO/+;+). Df(3) deletions were tested similarly. Thus, *para^bss1^/*Y;+;Df(3)/+ male flies were examined for enhancement, and *para^bss1^/+;+;Df(3)/+* flies were tested for suppression of BS phenotypes relative to their respective control siblings.

### Behavior and electrophysiology

Behavioral testing for BS paralysis was performed on flies 2−3 d after eclosion, as described previously (Kuebler and Tanouye 2000). Flies were anesthetized with CO_2_ before collection and tested the following day. For testing, 15−20 flies were placed in a food vial and stimulated mechanically with a VWR vortex mixer at maximum speed for 10 sec. For analysis, recovery time was measured for each fly from the end of the vortex stimulation until it resumed an upright standing position. Mean recovery time (MRT) was the average time taken for a fly exhibiting BS behavior to recover in a population. Pools of flies are combined (in total, n ≈ 100 for each genotype). For the purposes of comparisons, these are expressed here as normalized mean recovery time (nMRT), which is the MRT of the experimental flies divided by MRT of their control siblings. For genotypes that display only partial penetrance of BS paralysis, only those flies that displayed paralysis were used for recovery time analysis. A simpler measure of recovery time is RT_50_ (50% recovery time), the time at which half of BS flies have recovered from paralysis. RT_50_ was used in some analyses and especially to facilitate initial identification of enhancers and suppressors.

*In vivo* recording of seizure-like neuronal activity and seizure threshold determination in adult flies was performed as described previously (Kuebler and Tanouye 2000; Lee and Wu 2002). Flies 2−3 d posteclosion were mounted in wax on a glass slide, leaving the dorsal head, thorax, and abdomen exposed. Stimulating, recording, and ground metal electrodes were made of uninsulated tungsten. Seizure-like activity was evoked by high-frequency electrical brain stimulation (0.5-ms pulses at 300 Hz for 400 ms) and monitored by dorsal longitudinal muscle recording. During the course of each experiment, the giant fiber circuit was monitored continuously as a proxy for holobrain function. For each genotype tested, n ≥ 10, and unless otherwise noted, all flies were female. Comparisons of paralytic recovery time and seizure threshold were Student *t*-test. For all figures, error bars represent SEM, and statistical significance is indicated by *P < 0.01 and **P < 0.0001.

## Results

### Screening for *para^bss1^* enhancers with deficiencies

**Figure 1:**
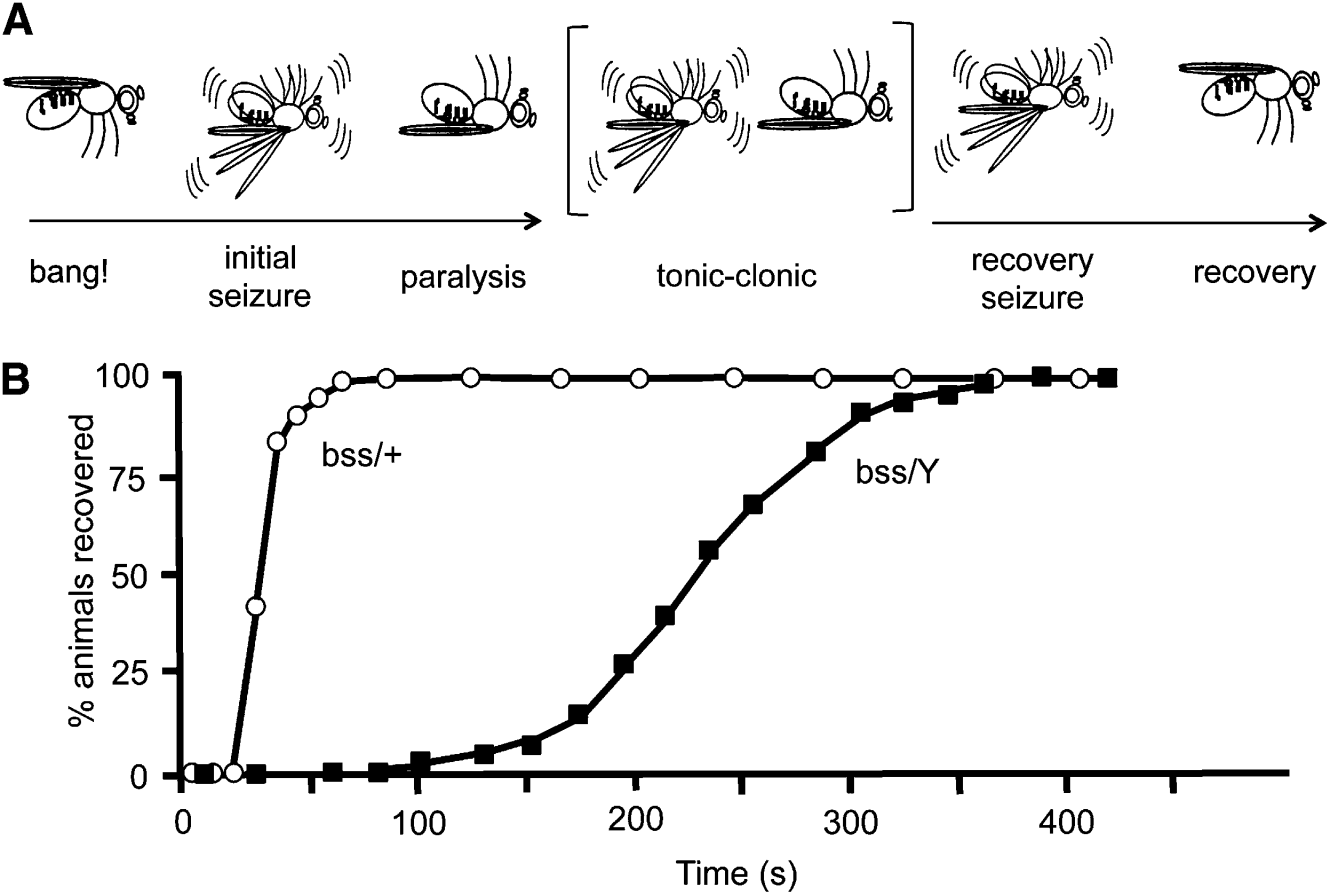
Behavior phenotypes for *para^bss1^* mutants. (A) Illustration depicting stereotype behavioral phenotype of *para^bss1^* flies subjected to a mechanical shock (10-sec vortex: “bang!”): initial seizure-like behavior, followed by complete paralysis and then a tonic/clonic period that is unique to *para^bss1^* and not evident in other BS mutant genotypes. One clonus-like event is depicted, but the number can vary, as can the duration of the period. The tonic/clonic-like period is followed by a recovery seizure, and the fly then recovers. Not depicted is a quiescent period of variable duration often observed between the recovery seizure and recovery, as well as the refractory period during which flies are resistant to further seizures that occurs immediately following recovery. (B) Recovery times from behavioral paralysis for *para^bss1^/Y* hemizygous males (labeled “bss/Y”) is substantially longer than for *para^bss1^/+* heterozygous females (labeled “bss/+”). For the enhancer screen described in the text, heterozygous deletions were selected that prolonged the *para^bss1^/Y* recovery time compared to sibling controls. For the suppressor screen described in the text, heterozygous deletions were selected that reduced the percentage of *para^bss1^/+* females paralyzed by the mechanical shock compared to sibling controls. (Figure adapted from Parker *et al.* 2011a).

**Figure 2:**
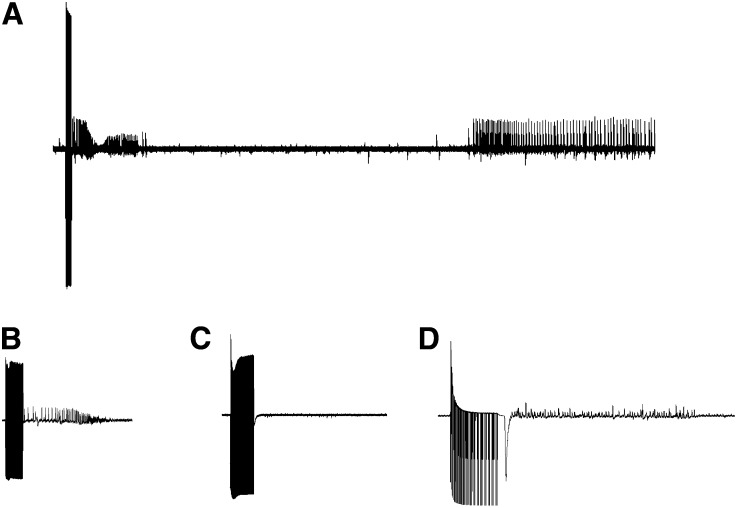
Electrophysiology phenotype of *para^bss1^* mutants. Seizure-like electrical activity in *para^bss1^* and wild-type flies. The mutant fly is more susceptible to seizures and has a lower threshold. (A) Seizure-like activity displayed at a slow sweep speed showing initial seizure, period of synaptic failure, and recovery seizure. (B) Seizure-like activity is evoked by 4-V HFS stimulus and displayed at high sweep speed. The mutant is susceptible to low-voltage evoked seizures indicating extreme seizure-sensitivity. (C) A low-voltage 4 V HFS stimulus delivered to a wild-type fly is ineffective at eliciting seizure-like activity because it is below the seizure threshold. (D) A greater voltage 30-V HFS stimulus delivered to a wild-type fly elicits seizure-like activity because it is above threshold for seizure initiation.

The *para^bss1^* mutant displays phenotypes that are similar to other mutants of the BS paralytic class such as *eas^PC80^*, *sda^iso7^*^.8^, and *tko^25t^* (Ganetzky and Wu 1982; Royden *et al.* 1987; Pavlidis *et al.* 1994; Zhang *et al.* 2002), albeit more severe. BS seizure-like behaviors and paralysis are observed in response to mechanical shock (“a bang”) (Figure 1). The time of BS paralysis for *para^bss1^* is much longer than for other mutants and exhibits unusual tonic-clonic-like behaviors. For example, total paralytic time for *para^bss1^* is about 240 sec, longer than for *sda^iso7.8^*, which is about 25 sec (Zhang *et al.* 2002; Parker *et al.* 2011a). The *para^bss1^* mutant also has a low threshold for seizure-like activity evoked by high-frequency electrical stimulation (HFS) of the brain. For example, seizure threshold for *para^bss1^* is 3.2 ± 0.6 V HFS, lower than the threshold for *sda^iso7.8^*, which is 6.2 ± 0.8 V HFS; wild-type Canton-Special flies have a seizure threshold of 30.1 ± 3.8 V HFS, for comparison (Figure 2) (Kuebler *et al.* 2001).

Despite the existing severity of *para^bss1^* phenotypes, we explored the possibility that these might be exacerbated further by enhancer mutations. We have previously found that recovery time from BS paralysis for *para^bss1^* varies with genetic background, age, and other factors (parker *et al.* 2011a). The length of time required for recovery appears to be primarily dependent on the number of bouts of tonic-clonic-like activity. We exploited this in an initial screen, investigating the possibility that potential enhancers may reside in chromosomal segments made haploid by deletions, and these would become manifest by a change in the time required to recover from BS paralysis. We then examined enhancers for effects on other *para^bss1^* phenotypes. We measured BS paralytic recovery times in *para^bss1^/Y*; *Df/+* flies compared with their control siblings of genotype *para^bss1^/Y*; *Balancer/+* (Table 1, File S1). Several deficiency chromosomes consistently showed increased recovery times for *para^bss1^* males (Table 1). For example, Df(2R)Exel7135 had a MRT of 363 s for experimental males, compared with 234 sec for their sibling controls yielding an nMRT of 1.55. Other notable deficiencies included: Df(2R)Exel6078 and Df(2R)Exel6056 with nMRTs of 2.27 and 2.53, respectively. Here we focus on Df(2R)Exel7135 as representative of our findings on *para^bss1^* enhancers.

**Table 1:** Chromosomal deletions that enhance the behavioral bang-sensitive (BS) paralytic phenotype of *para^bss1^/+* flies

Deficiency	Experimental (Df) MRT (s)	Control (Balancer) MRT (s)	nMRT
Df(2R)Exel7135	363	234	1.55
Df(2R)Exel6078	306	135	2.27
Df(2R)Exel7094	232	102	2.27
Df(2R)Exel6071	217	118	1.84
Df(2R)Exel6056	215	85	2.53

Values of the length of time that hemizygous *para^bss1^/Y* males remained paralyzed are depicted as MRT. To minimize the effects of genetic background, experimental males of the general genotype: *para^bss1^/Y;Df/+* were compared directly with sibling control brothers arising from the same cross (genotype: *para^bss1^/Y;Balancer/+*). The ratio of MRT for experimental males with that of their control siblings is listed as nMRT. MRT, mean recovery time; nMRT, normalized mean recovery time.

### Reduced expression of *charlatan (chn)* contained in the Df(2R)Exel7135 chromosomal segment enhances *para^bss1^* BS paralysis but not seizure threshold

**Figure 3:**
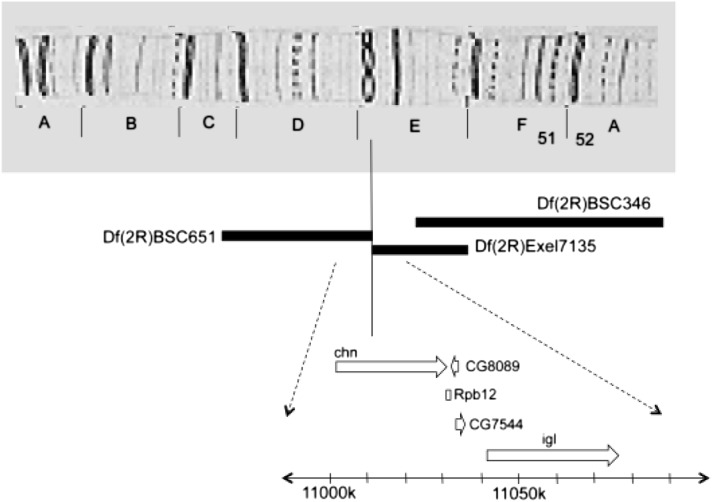
Chromosomal segment deleted in *Df(2R)Exel7135*. The upper panel of the figure depicts region 51 of the polytene chromosome. The *chn* gene is disrupted by the distal breakpoint of *Df(2R)BSC651* and the proximal breakpoint of *Df(2R)Exel7135*; both rearrangements enhance BS paralytic recovery time in *para^bss1^* hemizygotes. The BS paralytic recovery time phenotype is not enhanced by the *Df(2R)BSC346*.

The Df(2R)Exel7135 deficiency is a deletion spanning from 51E2 to 51E11 on chromosome 2R and contains approximately 22 genes. Deletion analysis further limited this segment to 51E2 to 51E7 on the basis of observations that the *para^bss1^/Y* recovery time is not enhanced by the heterozygous Df(2R)BSC346/+ (51E7-52C2) but is enhanced by Df(2R)BSC651/+ (51C5-51E2) (Figure 3, File S4). We found that BS enhancement in the segment is accounted for by reduced expression of the *charlatan (chn)* gene. The gene is broken by the 51E2 breakpoints of Df(2R)Exel7135 and Df(2R)BSC651 and is the only apparent gene affected by both rearrangements. Further identification of *chn* as an enhancer of *para^bss1^/Y* is by *UAS-chnRNAi*. Flies of the genotype *ELAV-Gal4^C155^ para^bss1^/Y;UAS-chnRNAi/+* show increased BS recovery times with an MRT of 261.9 ± 17.1 sec compared with 105.6 ± 9.4 sec for their *ELAV-Gal4 para^bss1^/Y;+/+* sibling controls for an nMRT of 2.48 (*P* < 0.001) (File S4).

The *chn* gene encodes an NRSF/REST transcriptional repressor of neuronal-specific genes (Escudero *et al.* 2005; Tsuda *et al.* 2006; Yamasaki *et al.* 2011). It has not been previously identified in seizure susceptibility or electrical excitability. Surprisingly, the enhancement of *para^bss1^* by *chn* was limited to BS paralysis recovery time phenotype, that is, an increase in the severity of this phenotype; there was no apparent enhancement of the other major phenotype: threshold for evoked seizure. For example, flies of the genotype *ELAV-Gal4^C155^ para^bss1^/Y;UAS-chnRNAi/+* have a seizure threshold of 3.32 ± 0.47 V HFS, similar to the threshold of 3.87 ± 0.53 V HFS (*P* = 0.46) for their sibling controls (File S3). Flies of the genotypes *ELAV-GAL4^C155^/Y;UAS-chnRNAi/+* and Df(2R)Exel7135*/Cyo* exhibited no bang sensitivity, indicating that *chn* enhances seizure severity without being a bang-sensitive mutant itself (File S4). These findings are consistent with the results of Df(2R)Exel7135 and all of the other enhancers identified in this screen: the enhancers increased BS paralysis time to recovery but did not reduce seizure threshold in electrophysiology tests.

### Screening for *para^bss1^* suppressors with deficiencies

The *para^bss1^* mutant is severely seizure sensitive: phenotypes are difficult to suppress by antiepileptic drug feeding and Drosophila seizure-suppressor mutations thus far identified have been ineffective at alleviating *para^bss1^* phenotypes. The *para^bss1^* mutation is semidominant with seizure-like behaviors, and BS paralysis reduced in heterozygous *para^bss1^/+* flies, but still present at high penetrance (>95%) (Figure 1) (Ganetzky and Wu 1982; Parker *et al.* 2011a). We exploited this feature to screen for suppressor mutations inferring that heterozygotes would provide a genetic background that is sensitized for detecting putative suppressors. As an initial screen, we investigated the possibility that potential suppressors may reside in chromosomal segments made haploid by deletions and that these would become manifest by a change in BS paralysis. That is, we compared *para^bss1^/+*; *Df/+* females with their control sisters of genotype *para^bss1^/+*; *Balancer/+* for differences in the percentage of flies undergoing BS paralysis. Several deletion chromosomes consistently reduced the BS phenotype in *para^bss1^*/+ females (Table 2, File S1). For example, only 13% of *para^bss1^/+*; *Df(3R)ED10639/+* females showed BS paralysis compared with their sibling controls, an apparent phenotypic suppression of approximately 87%. Other notable deletions included *Df(2R)Exel6285* and *Df(3L)ED4502* that caused 97% and 93% suppression, respectively. Here, we focus on *Df(3R)ED10639* as representative of our findings on *para^bss1^* suppressors.

**Table 2:** Chromosomal deletions that revert the behavioral bang-sensitive (BS) paralytic phenotype of *para^bss1^/+* flies

Deficiency	BS
Wild type	0.00
Df(2R)Exel6285	0.03
Df(3L)ED4502	0.07
Df(3R)ED10639	0.13
Df(3L)ED224	0.19
Df(3L)ED201	0.29
Df(3L)ED4502	0.42
Df(2R)BSC427	0.49
Df(3R)ED5518	0.50
Df(3L)ED4486	0.50
*para^bss1^/+*	0.95

Ordinarily, approximately 95% of *para^bss1^/+* flies show a BS paralytic phenotype: paralysis aftermechanical stimulation. Wild-type flies never show BS paralysis. The number of flies showing BS paralysis is greatly reduced by the deficiency chromosomes listed in the table. Flies tested carried the heterozygous deficiency and were of the general genotype: *para^bss1^/+*; *Df/+*. In all cases, to control for genetic background, experimental flies were compared directly with sibling control flies arising from the same cross (genotype: *para^bss1^/+*; *Balancer/+*).

### Reduced expression of *gilgamesh (gish)* contained in the *Df(3R)ED10639* chromosomal segment suppresses *para^bss1^*/+ BS paralysis

**Figure 4:**
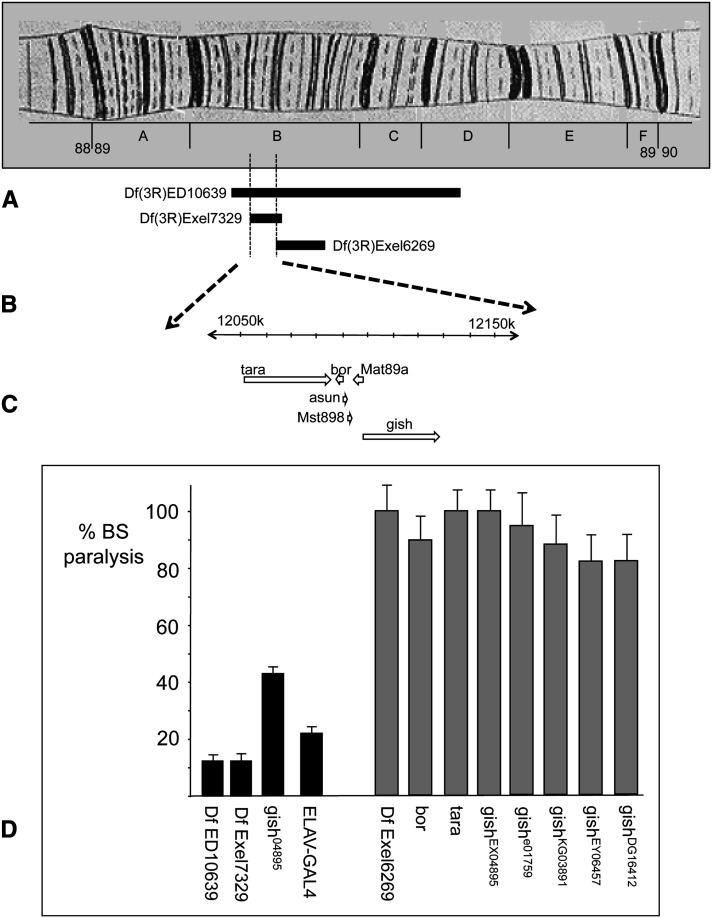
Suppression of *para^bss1^*/+ BS paralytic phenotype by a heterozygous chromosomal segment deleted in 89B. (A) Depicted is polytene chromosome map of region 89 on 3R. (B) The segment deleted in *Df(3R)ED10639* causes suppression of *para^bss1^/+* BS paralysis, as described in the text. Also, *Df(3R)Exel7329* causes suppression but *Df(3R)Exel6269* does not. The breakpoints of these rearrangements delimit a small region (89B9 to 89B12) responsible for seizure suppression. (C) Six genes are contained in the 89B9 to 89B12 chromosomal segment including *tara*, *bor*, and *gish*. (D) BS paralytic phenotypes (% BS paralysis) of several genotypes in a *para^bss1^*/+ background, as described in the text. Genotypes showing BS suppression are depicted as black bars; gray bars are used in genotypes showing no suppression. In each case, the experimental genotype shown is normalized relative to sibling controls. Df ED10639 is the genotype *para^bss1^*/+; *Df(3R) ED10639/+* showing 13% BS paralysis (87% suppression of BS phenotype). This indicates the apparent presence of a gene that acts as a haplo-seizure suppressor. Df Exel7329 is *para^bss1^*/+;*Df(3R)Exel7329/+* showing 13% BS paralysis and providing one boundary for suppressor location at 89B9 based on inclusion within the deleted segment. Df Exel6269 is *para^bss1^*/+;*Df(3R)Exel6269/+* showing 100% BS paralysis and providing a second boundary for suppressor location at 89B12 based its exclusion from the deletion. Flies that are *para^bss1^*/+;*bor^c05496^/+* and *para^bss1^*/+;*tara^1^/+* (labeled bor and tara) show no suppression with 91% and 100% BS paralysis, respectively. Flies that are *para^bss1^*/+;*gish^04895^/+* (labeled gish^04895^) show 43% BS paralysis, indicating suppression of the BS paralytic phenotype. Flies that are *para^bss1^*/+;*gish^EX04895^/+* (labeled gish^EX04895^) are a line with a remobilized, precise excision of the *gish^EX04895^* P-element; they show no suppression with 98% BS paralysis. Flies that are *ELAV-Gal4^C155^ para^bss1^*/+; *UAS-gishRNAi/+* (labeled ELAV-GAL4) show 25% BS paralysis indicating suppression of the BS paralytic phenotype. Several *gish* alleles as heterozygotes show no suppression of *para^bss1^*/+ BS paralytic phenotypes. Thus, gish^e01759^/+, gish^DG16412^/+, gish^KG03891^/+, gish^EY06457^/+ heterozygous combinations in a *para^bss1^*/+ background show 95%, 88%, 84%, and 83% BS paralysis, respectively.

The *Df(3R)ED10639* deficiency is a deletion spanning from 89B7 to 89D5 and contains approximately 57 genes. In this section, we describe analyses showing that *para^bss1^*/+ suppression in the segment is accounted for by reduced expression of the *gilgamesh (gish)* gene (Figure 4). *para^bss1^*/+ BS suppression phenotype was mapped to a small region on chromosome 3R between 89B9 and 89B12 using overlapping deficiencies. In particular, localization of the suppression phenotype is based on its inclusion in the *Df(3R)Exel7329* deletion, which affects the number of animals paralyzed (Figure 4) (89B9-89B13), and its exclusion from the *Df(3R)Exel6269* deletion which has no effect on paralysis (Figure 4) (89B12-B18). This localization is consistent with the combined findings from other overlapping deletions in the region (Figure 4).

The 89B9-89B12 segment contains six genes (Figure 4). We found that an allele of *belphegor (bor)*, *para^bss1^/+;bor^c05496^/+*, which showed similar BS paralysis compared with control siblings (9% reduction in BS paralysis), did not appear to cause suppression based on flies of the genotype: Also, an allele of *taranis (tara)* did not appear to cause suppression based on flies of the genotype *para^bss1^/+;tara****^1^****/+*, with BS paralysis similar to their sibling controls (0% reduction in BS paralysis). In contrast, an allele of *gilgamesh (gish)* caused substantial suppression based on flies of the genotype *para^bss1^/+;gish^04895^/+*, which showed a 57% reduction in BS paralysis compared with their *para^bss1^/+;TM3/+* control siblings (File S4).

### The *gish* gene

The *gish* gene of Drosophila is homologous to mammalian casein kinase CK1g3, both members of the CK1 family of serine-threonine kinases (Zhai *et al.* 1995). The Drosophila gene is approximately 30 kb and alternatively spliced to express 12 different isoforms in four main classes (Hummel *et al.* 2002; Tan *et al.* 2010). These arise from two initiation sites: two classes of long transcript (~3 kb) arise from an upstream initiation site; two classes of short transcript (~2.5 kb) from a downstream initiation site (Hummel *et al.* 2002; Tan *et al.* 2010). The *gish^04895^* mutation is a *P*-element insertion in exon 2, present in long, but not short *gish* transcripts. Reverse-transcription polymerase chain reaction analysis (Tan *et al.* 2010) has shown that long *gish* transcripts are apparently undetectable in *gish^04895^* mutants. Interestingly, in contrast, short transcripts appear to be more abundant in *gish^04895^* mutant than in wild-type flies (Tan *et al.* 2010). In the present experiments, *gish^04895^* acts as a recessive lethal, in contrast to previous reports, suggesting that it is a viable (Tan *et al.* 2010). We are unclear on the reasons for this apparent difference in viability. We find that precise excision of the *gish^04895^ P*-element completely reverted the BS suppressor phenotype (Figure 4, File S2, File S4), restored viability, but did not appear to revert the male sterility phenotype seen among *gish* mutant alleles (Castrillon *et al.* 1993).

Identification of *gish* as a *para^bss1^/+* BS suppressor by mutant analysis was supported further by RNAi analysis. Flies of the genotype *ELAV-Gal4^C155^ para^bss1^/+;UAS-gishRNAi/+* showed a 75% reduction in BS paralysis compared with their *ELAV-Gal4^C155^ para^bss1^/+;+/+* control siblings, showing that BS suppression occurred when *gish* expression was reduced in all neurons with the *ELAV-Gal4* pan-neuronal driver (File S4). We propose that *gish* is a suppressor of *para^bss1^/+* based on reversion of phenotypes by *gish^04895^/+*, by *ELAV-Gal4^C155^-driven UAS-gishRNAi*, by *Df(3R)ED10639/+*, and by *Df(3R*)*Exel7329/+*. Several mutant alleles of *gish* that failed to suppress *para^bss1^/+* BS paralytic phenotypes were also found in these analyses. Thus, suppression was not observed for 3 *P*-element mutations with inserts in the second intron of *gish* which is spliced out of the long transcripts (genotypes: *para^bss1^/+;gish^KG03891^*, *para^bss1^/+;gish^DG16412^*, and *para^bss1^/+*;*gish^EY06457^*) (Figure 4). No suppression was seen in *para^bss1^/+;gish^e01759^/+* flies, which has an insert upstream of the first transcript initiation site (Figure 4, File S4).

### The *gish^04895^* mutation raises the threshold for evoked seizures in *para^bss1^*/+ flies

**Figure 5:**
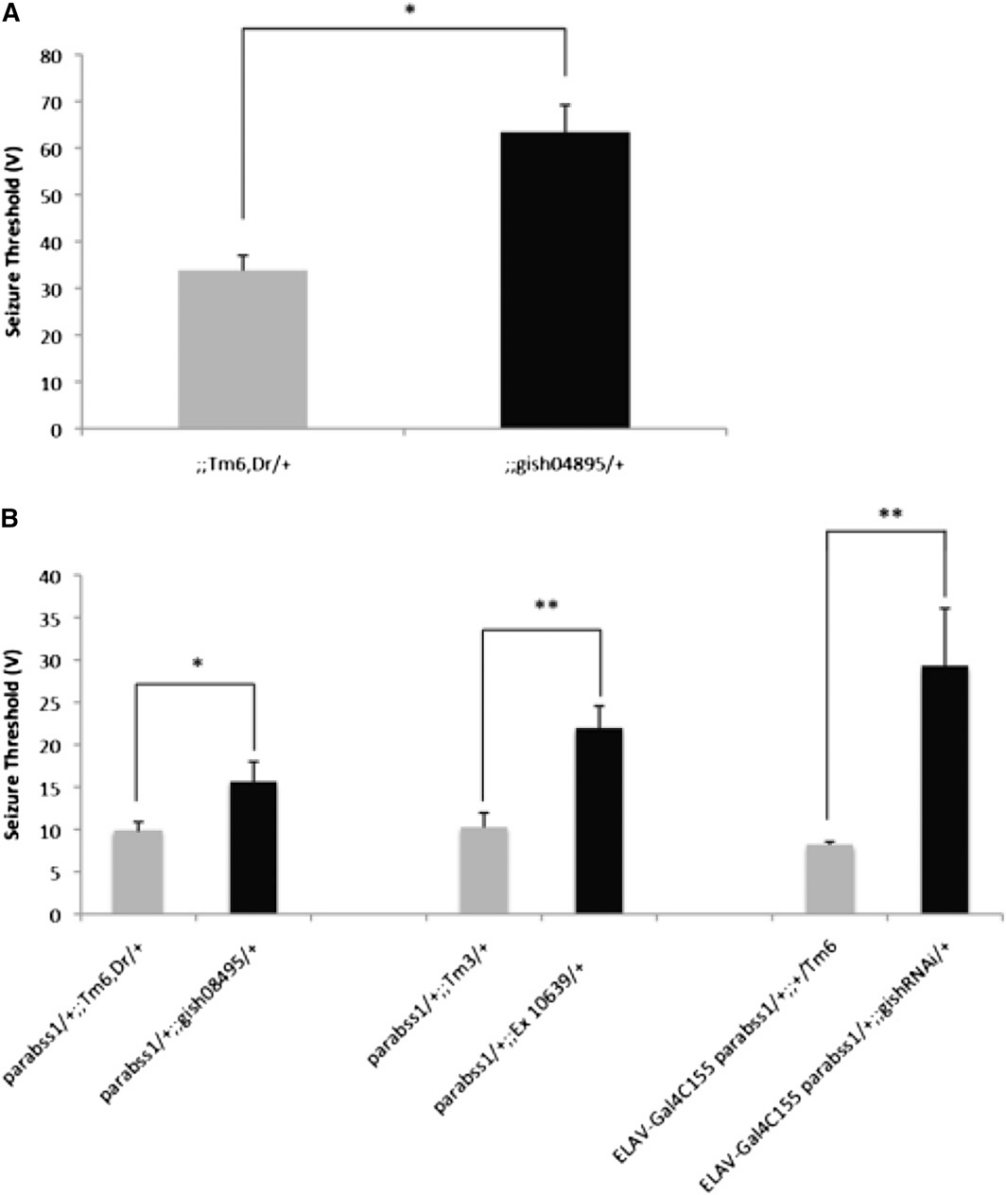
Suppression of seizure threshold by *gish^04895^* and Df Ed10639. Seizure-like activity was recorded in flies of different genotypes. Depicted are the relative HFS voltages required to evoke seizure-like activity at threshold. Loss-of-function mutations of *gish* suppress seizure-sensitivity in *para^bss1^* heterozygotes, indicated by an increase in seizure threshold voltage compared to controls. In each case, experimental flies are compared with controls that are siblings arising from the same cross in order to minimize genetic background differences. (A) Seizure threshold of *gish^04895^/+* compared with the wild type. The heterozygous mutant *gish^04895^/+* has a slightly greater voltage at threshold suggesting that it is a seizure-resistant mutation. (B) Seizure thresholds of *para^bss1^* heterozygotes in different seizure-suppressor backgrounds. Experimental *gish^04895^/+* flies were of the genotype *para^bss1^/+*; *gish^04895^/+* and had a greater seizure threshold than their control siblings (genotype: *para^bss1^/+*; *TM6,Dr/+*), indicating seizure-suppression. Experimental *Df Ed10639/+* flies were of the genotype *para^bss1^/+*; *Df(3R)Ed10639/+* and had a greater seizure threshold than their control siblings (genotype: *para^bss1^/+*; *TM3/+*) indicating seizure suppression. Experimental *ELAV-Gal4*-driven *gish*RNAi flies were of the genotype ELAV-Gal4^C155^ *para^bss1^/+*; *UAS-gishRNAi/+* and had a higher seizure threshold than their control siblings (genotype: *ELAV-Gal4^C155^ para^bss1^/+*; *TM6/+*) indicating seizure-suppression.

The mutation *gish^04895^* is a recessive lethal. As a heterozygote, in a wild-type background, it displays a seizure-resistant phenotype. Thus, the seizure threshold of *gish^04895^/+* flies is about twice that of wild-type Canton-Special flies, 63.4 ± 5.8 V HFS and 33.8 ± 3.2 V HFS, respectively (Figure 5). The *gish^04895^/+* flies have no other apparent phenotypes: their electrophysiology, behavior, and morphology are all wild type.

Seizure-suppression for *gish* is seen with flies of the genotype: *para^bss1^/+*; *gish^04895^*/+, which show a seizure threshold of 15.6 ±2.42 V HFS, which is greater than the threshold of their *para^bss1^/+;TM6*/+ control siblings (9.8 ± 1.09 V HFS seizure threshold; Figure 5). This seizure-suppression is caused by a loss of *gish* function as seen most clearly in deletion flies: *para^bss1^/+;Df(3R)ED10639*/+ show a seizure threshold nearly in the wild-type range (22.0 ± 2.62 V HFS; Figure 5). Their *para^bss1^/+;TM3*/+ siblings show a low seizure threshold (10.3 ± 1.73 V HFS). The loss of *gish* function finding was confirmed further by RNAi analysis. Flies of the genotype *ELAV-Gal4^C155^ para^bss1^/+*; *UAS-gishRNAi/+* showed an increased seizure threshold of 29.28 ± 6.78 V HFS compared with their *ELAV-Gal4^C155^ para^bss1^/+*; *+/Tm6* control siblings (8.19 ± 0.355 V HFS; Figure 5, File S3).

### Seizure suppression by *gish* is specific to *para^bss1^*/+ heterozygotes

**Figure 6:**
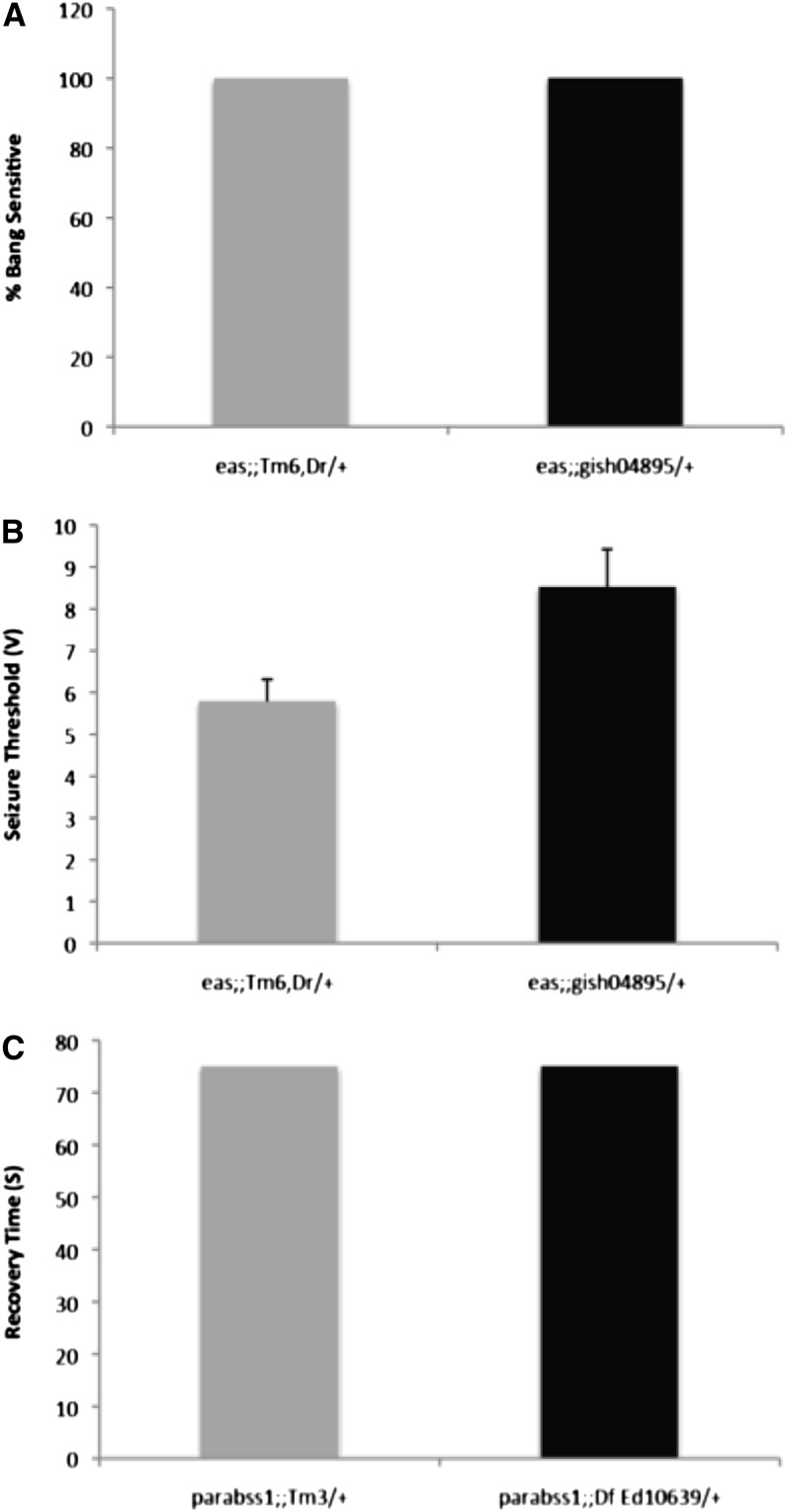
Suppression of seizure sensitivity by *gish* is specific to *para^bss1^* heterozygotes. (A) The percentage of *eas* flies showing a bang sensitive paralytic behavioral phenotype is not reduced by *gish^04895^*/+. Paralysis is 100% of flies in both experimental (genotype: *eas*; *gish^04895^*/+) and control siblings (genotype: *eas*; *TM6*, *Dr/+*) genotypes. (B) Electrophysiological recording shows that the seizure threshold of *eas* is a little greater in a *gish^04895^*/+ background (genotype: *eas*; *gish^04895^*/+), but there is no significant suppression compared with control siblings (genotype: *eas*; *TM6*, *Dr/+*). (C) Recovery time of *para^bss1^* homozygotes and hemizygotes is not altered by *gish* loss-of-function. Depicted are recovery times compared between *para^bss1^*; *Df(3R)Ed10639/+* experimental flies and their control siblings (genotype: *para^bss1^*; *TM3/+*).

Seizure suppressor mutations that have been identified previously have been general suppressors, each suppressing several Drosophila BS mutants. In contrast, *gish^04895^*/+ suppression is found here to be specific: it appears to only suppress *para^bss1^*/+ heterozygotes. We tested for *gish^04895^/+* suppression against BS mutant, *eas*: *gish* was ineffective as a suppressor. Thus, *eas* mutants showed 100% BS paralysis in a *gish^04895^*/+ background; electrophysiology also showed minimal increases in seizure threshold (Figure 6, File S3, File S4). We also find that *gish/+* does not suppress phenotypes of *para^bss1^* homozygous females and *para^bss1^*/*Y* hemizygous males. Thus, *para^bss1^* homozygotes and hemizygotes showed 100% BS paralysis in a *gish* background: BS paralysis could not be suppressed by *gish^04895^/+*, by *Df(3R)ED10639*/+, or by *UAS-gishRNAi*. In addition, a *Df(3R)ED10639*/+ background caused no reductions of BS paralytic recovery time in *para^bss1^* homozygotes and hemizygotes, a phenotype of *para^bss1^* that is ordinarily easier to suppress than BS paralysis (Figure 6, File S4).

### Seizure suppression by *gish* does not appear to be dependent on Wg/Wnt signaling

The *prickle* gene functions in noncanonical Wg/Wnt signaling, and mutations have been found to cause myoclonic seizures in humans and BS paralytic behavior in Drosophila (Tao *et al.* 2011). CK1g casein kinases subserve a large number of cellular processes with diverse substrates (Knippschild *et al.* 2005), and one prominent role for *gish* is to phosphorylate *arrow*, a co-receptor for Wg (Zhang *et al.* 2006). To test whether seizure suppression by *gish* might be via Wg signaling, we examined other components of the pathway by RNAi. To test *arrow* loss-of-function, flies of the genotype *ELAV-Gal4^C155^ para^bss1^/+*;; *UAS-arrRNAi/+* showed a slightly lower, but not significant percentage of BS paralysis compared with control *ELAV-Gal4^C155^ para^bss1^/+*; *+/Tm6* flies (data not shown, File S4). To test *Wg* and *pangolin* loss-of-function, flies of the genotypes *ELAV-Gal4^C155^ para^bss1^/+*; *UAS-WgRNAi/+* and *ELAV-Gal4^C155^ para^bss1^/+*; *UAS-panRNAi/+* were comparatively equal in percentage of BS paralysis as their *ELAV-Gal4^C155^ para^bss1^/+*; *tft/+* controls (data not shown, File S4). Thus, we conclude that seizure suppression by *gish* is not directly linked to Wg/Wnt signaling.

## Discussion

In the present article, we examine severe seizure phenotypes and explore the possibility that severity may be modulated by genetics. We use as substrate the Drosophila *para^bss1^* mutation a channelopathy affecting the voltage-gated Na^+^ channel. Severe seizure sensitivity is observed in *para^bss1^* mutants, severity that is unresponsive to available drug treatment. In addition, *para^bss1^* has not responded to seizure suppressor mutations identified in screens based on the Drosophila mutants *eas* and *sda*. The present study is based on an unbiased, forward genetics screen for mutations that interact with *para^bss1^* by either exacerbating seizure phenotypes (seizure enhancer mutations) or reducing the severity of phenotypes (seizure suppressor mutations).

The search for *para^bss1^* enhancers and -suppressors identified several candidates. Analysis of *chn* was representative of an enhancer. We found that the time of paralysis of *para^bss1^* individuals was increased (the phenotype screened for), but there was otherwise no obvious enhancement of seizure-sensitivity or severity. Behavioral phenotypes of *para^bss1^* generally resemble those of other BS mutants: all BS mutants are behaviorally similar in initial seizure, initial paralysis, and recovery seizure (Parker *et al.* 2011a). Unlike other BS mutants, initial paralysis in *para^bss1^* homozygotes is followed by an extended period of tonic/clonic-like activity, resembling activity observed in several human epilepsies (Parker *et al.* 2011a). During this period in *para^bss1^*, the fly is mainly quiescent, resembling a tonic phase. The quiescence is broken up by multiple bouts of clonus-like activity. Because of its period of tonic/clonic-like activity, *bss^1^* recovery time is much longer than for other BS mutants such as *sda or eas* (Parker *et al.* 2011a). It is this recovery time, the tonic/clonic period, that is extended by the *chn* enhancer mutation. A surprise to us was that there was no *chn* enhancement of the other major *para^bss1^* phenotype: a low electrophysiology seizure threshold. Also, the *chn* mutation is the only seizure enhancer that we have identified thus far, that does not cause any BS phenotypes (Glasscock and Tanouye 2005; Hekmat-Scafe *et al.* 2006).

Analysis of *gish* was representative of a *para^bss1^* suppressor. We found that seizure sensitivity of heterozygous *para^bss1^/+* individuals was greatly reduced by *gish* loss-of-function mutation and by RNAi. Also, electrophysiological threshold is increased, a further indication that seizure-susceptibility has been reduced in *para^bss1^/+* individual flies. The *para^bss1^* mutant has been exceptionally difficult to suppress. Previously, we have identified 13 seizure-suppressor mutations that suppress the BS behavioral phenotypes of *sda* and *eas* mutants, and raise the electrophysiology seizure threshold, often to nearly wild-type levels (reviewed in Parker *et al.* 2011b). However, seizure suppressors identified heretofore have been ineffective at suppressing *para^bss1^* phenotypes. Seizure suppression by *gish* loss-of-function mutations reported here is unusual in several respects. It is the only seizure suppression that is effective in reverting *para^bss1^* phenotypes, although it is effective only with heterozygotes, and not homozygotes or hemizygotes. Surprisingly, the seizure suppression is ineffective with *sda* and *eas* mutants. Previously, we had attributed this simply to different seizure-sensitive mutants being more or less refractory to suppression. The present results suggest, however, that there may be a fundamental difference between *sda* and *eas* mutants, on the one hand, and *para^bss1^* on the other. The nature of the difference remains unclear, at present, but *para^bss1^* seems somehow to be special. We suspect that this could be because of something special about the voltage-gated Na^+^ channel, the gain-of-function nature of the *para^bss1^* mutation, or both. Also, somewhat perplexing is the reason why *para^bss1^* phenotypes might be suppressed by *gish* mutations, how a loss of casein kinase function can interfere with voltage-gated Na^+^ gain-of-function. Also, we do not yet know whether the presence of normal Na^+^ function (in the heterozygote) is a strict requirement for the suppression of the gain-of-function mutation. The *gish* mutations do not appear to otherwise be seizure-suppressor mutations, as judged by their lack of effectiveness with *sda* and *eas*, but their suppression of *para^bss1^* is pretty remarkable.

It is clear from this study that *gish* is capable of suppressing *para^bss1^/+* phenotypes and from other deletions identified in our screen that additional suppressor mutations may be found. The *para^bss1^* mutant has been presented as a model for human intractable epilepsy, especially Dravet syndrome (Dravet 1978), a Na^+^ channelopathy (Parker *et al.* 2011a). The findings presented here on *gish* suppression of *para^bss1^* suggest a compelling novel approach for developing options for intractable epilepsy therapeutics depending on exactly how well *para^bss1^* models Dravet syndrome or other intractable epilepsies and how well these findings transfer to mammalian models. At present, available data show that the *para^bss1^* model is a good one. Further experiments of this type as well as the isolation of new suppressors may bring us closer to unraveling the complexity of seizure disorders, especially intractable disorders.

## Supplementary Material

Supporting Information
