# Supplementary material for: Drosophila as a Model for Intractable Epilepsy: Gilgamesh Suppresses Seizures in parabss1 Heterozygote Flies
Source: G3 (Bethesda). 2013 Aug 1;3(8):1399–407. doi: 10.1534/g3.113.006130 (PMC3737179; doi:10.1534/g3.113.006130)
Supplement: Supporting Information [file supp_3_8_1399__index.html]

Drosophila as a Model for Intractable Epilepsy: Gilgamesh Suppresses Seizures in parabss1 Heterozygote Flies — Supporting Information 

# *Drosophila* as a Model for Intractable Epilepsy: *Gilgamesh* Suppresses Seizures in *parabss1* Heterozygote Flies

## Supporting Information for Howlett *et al.*, 2013

**Files in this Data Supplement:**

- Supporting Information - Files S1-S4 (PDF, 372 KB)
- File S1 - Screen Data (PDF, 160 KB)
- File S2 - Sequence Data (PDF, 101 KB)
- File S3 - Seizure Threshold Data (PDF, 173 KB)
- File S4 - Bang-Sensitivity Data (PDF, 129 KB)
