# Supplementary material for: Drosophila as a Model for Intractable Epilepsy: Gilgamesh Suppresses Seizures in parabss1 Heterozygote Flies
Source: G3 (Bethesda). 2013 Aug 1;3(8):1399–407. doi: 10.1534/g3.113.006130 (PMC3737179; doi:10.1534/g3.113.006130)
Supplement: Supporting Information [file supp_g3.113.006130_006130SI.pdf]

G3 · Genes | Genomes | Genetics

Drosophila as a model for intractable epilepsy: *gilgamesh* suppresses seizures in *para*^{bss1} heterozygote flies

Iris C. Howlett[*], Zeid M. Rusan[*], Louise Parker[**], Mark A. Tanouye[*,**]

*Department of Molecular and Cell Biology
University of California
Berkeley, CA 94720

**Department of Environmental Science, Policy and Management
University of California
Berkeley, CA 94720

DOI: 10.1534/g3.113.006130

File S1

Screen Data

Stock (Bloomington)	Chromosome	Df BS%	Bal BS%	Rec time Df	Rec time Bal	Difference
7532	2	50	32	73	94	21
7533	2	96	68	155	152	-3
7534	2	96	90	172	210	38
7535	2	96	84	114	120	6
7536	2	40	16	95	115	20
7537	2	86	55	97	127	30
7538	2	76	51	215	85	-130
7539	2	92	70	160	177	17
7540	2	88	75	90	82	-8
7541	2	89	90	171	191	20
7543	2	86	53	73	126	53
7544	2	91	75	132	129	-3
7545	2	72	48	131	159	28
7546	2	98	71	205	190	-15
7547	2	45	42	85	116	31
7548	2	71	59	90	106	16
7549	2	76	47	131	132	1
7551	2	89	82	143	126	-17
7553	2	98	69	217	118	-99
7554	2	54	18	128	108	-20
7556	2	98	65	216	129	-87
7557	2	94	85	186	216	30
7558	2	55	44	306	135	-171
7559	2	76	85	284	215	-69
7561	2	78	65	172	215	43
7748	2	81	64	97	138	41
7749	2	94	53	77	123	46
7750	2	2	72	96	121	25
7858	2	95	81	163	184	21

7859	2	84	45	232	102	-130
7860	2	93	57	144	128	-16
7862	2	88	73	119	157	38
7863	2	97	70	143	221	78
7864	2	89	68	131	139	8
7867	2	92	72	103	105	2
7869	2	94	75	120	157	37
7870	2	92	34	146	204	58
7871	2	91	81	145	190	45
7872	2	82	30	198	136	-62
7873	2	92	95	142	214	72
7875	2	94	50	162	194	32
7876	2	94	44	106	111	5
7877	2	95	66	118	156	38
7879	2	86	47	264	164	-100
7880	2	91	81	161	174	13
7881	2	68	47	99	115	16
7882	2	63	16	99	99	0
7883	2	79	32	111	101	-10
7886	2	76	41	152	193	41
7887	2	86	51	114	99	-15
7888	2	93	45	204	149	-55
7890	2	75	6	118	120	2
7891	2	35	33	73	123	50
7893	2	94	73	144	161	17
7894	2	84	64	98	142	44
7895	2	42	8	88	106	18
7898	2	62	79	127	124	-3
7900	2	85	57	109	175	66
7901	2	98	83	203	160	-43
7902	2	82	63	104	147	43
7903	2	95	30	139	147	8
7906	2	91	89	192	239	47
7908	2	70	20	239	133	-106

7909	2	95	89	241	217	-24
7916	2	70	18	116	122	6
7998	2			107	113	6
9064	2	76	93			0
24931	2	48	98			0
8082	3	77	90	92	121	29
9481	3	11	82	76	75	-1
9482	3	76	85			0
7736	3	95	81			0
7987	3	94	81			0
7984	3	89	87			0
7597	3	73	79			0
25689	3	70	79			0
24140	3	51	82	124	112	-12
8962	3	95	98	115	88	-27
24143	3	87	68	83	79	-4
8065	3	82	69	116	102	-14
7623	3	47	61	256	248	-8
8047	3	53	99	140	136	-4
8048	3	100	99	122	135	13
9693	3	94	91	221	229	8
8053	3	96	99	164	135	-29
7562	3	99	99	108	120	12
8096	3	85	100	178	175	-3
24410	3	96	100	169	162	-7
7570	3	75	98	189	198	9
8059	3	71	96	159	160	1
7571	3	98	99			0
24415	3	96	100			0
9701	3	100	97	185	172	-13
24941	3	89	100	115	113	-2
8070	3	90	92	163	178	15
8069	3	65	95	74	67	-7
23668	3	77	99	108	111	3

8075	3	74	77	155	140	-15
8072	3	50	100	110	107	-3
8074	3	98	90	152	146	-6
8100	3	79	94	150	127	-23
8099	3	95	99	157	145	-12
8081	3	95	95	200	213	13
8080	3	18	95	110	114	4
2492	3	76	92	116	129	13
8082	3	89	96	119	130	11
24952	3	98	98	184	173	-11
8088	3	67	96	104	127	23
8101	3	85	83	145	113	-32
9700	3	98	96	162	154	-8
8967	3	82	96	101	82	-19
7623	3	88	90	319	306	-13
9629	3	100	100	223	226	3
24968	3	97	91	192	197	5
8682	3	83	83	96	77	-19
24990	3	93	68	125	89	-36
9080	3	93	77	74	80	6
9084	3	45	90	116	96	-20
9090	3	53	69	107	143	36
25019	3	97	99	150	162	12
24137	3	60	83	103	78	-25
6962	3	44	59	107	109	2
7983	3	89	95	190	175	-15
7737	3	96	95	109	116	7
9207	3	55	92	95	103	8
8104	3	53	85	114	88	-26
25014	3	78	91	119	132	13
9486	3	100	99	170	161	-9
24139	3	43	67	119	79	-40
8962	3	37	65	73	56	-17
24915	3	90	93			0

25001	3	96	83	123	144	21
8097	3	6	81	117	99	-18
7562	3	99	95	149	160	11
8047	3	22	77	126	88	-38
8048	3	90	93	107	82	-25
9693	3	97	94	154	148	-6
7570	3	86	93	91	98	7
8096	3	50	89	89	76	-13
8073	3	83	92	106	61	-45
24410	3	89	84	146	97	-49
7571	3	91	100	182	99	-83
24392	3	94	92	170	87	-83
8060	3	69	84	32	78	46
24941	3	78	89	152	116	-36
24915	3	86	95	160	102	-58
8061	3	80	50	101	59	-42
9701	3	97	99	149	100	-49
24413	3	99	98	190	134	-56
8065	3	77	88	137	90	-47
8974	3	94	94	170	98	-72
7929	3	94	94	156	116	-40
24415	3	81	82	124	91	-33
23668	3	80	89	107	94	-13
9355	3	70	82	92	81	-11
8066	3	76	87	120	95	-25
8068	3	72	83	56	75	19
8070	3	84	90	79	64	-15
8073	3	91	96	99	58	-41
8097	3	37	88	136	105	-31
8074	3	76	91	128	97	-31

File S2

Sequence Data

Red C is insertion point of P-element from Bloomington stock 11790. Blue letters are where primers were designed to verify presence of insert.

4/8/10 - Primers designed to be used with P-element inverse repeat primer. If insert is there, product should be seen following PCR.

- Gish Forward Primer – gish11790_f (to be used with Repeat Reverse Primer) - CGT AGC ACG AGG CTG TTT TC (expected size ~516)
- Gish Reverse Primer – gish11790_r (to be used with Repeat Forward Primer) - CGC AAG CCT ATG GTC TCT AG (expected size ~523)

Gilgamesh gene region sequence near insertion (from Flybase)

ATATGGCCAAAAGTCGAGTAGTTACACCCTCTCTGCCTCTTTGCCCCTCTGCCTTTCTTGGTTTTCTCTCTCT
TATTCTCTTTGGGTCTTTGGCCATCGCCGCCTTGGCTCGTTTTTTGCATCGAATAACAAAACTTGAAAATTTA
TAAAAATAAACGAAATCACGTACGGAAATATTTACTTCGACCTTGCGGCCCCAGAGATTTTTCCAACAATTTA
TACGGCGCGGTGGCCATGGCAGCTAATTAGCCTAATTTTCTGTAATTTTCTAAATTTTAATTTGGTACCTTTTA
GAAGAGAGTAACTTTCATTTGTGCGACGATGACTTTAAAAGTTTTAAGTTAAAGTCAATAGTAACTCTTACTAA
ATTAAATGAAAATCAATTGAAAAAAAAAAAACAAATTTAGTTAGCGCTCTCATCAGTGCTTTTAGTGTGACCAG
CATACGCTATGGCTGGCCTAACATACCAAAAGAAATACCGCAGTTTCGTACATTCATTAGTCTATTTACTATT
TTTATTATCCATACAGAATGAAAAATGTTTAAATCGTCGGTAATATGAGTTTTTTTATATTTTGTATCAGTTAAC
ATGATCTTTCGTCAAAACATTCAAAGCGCTTGGAATTAAGACCGATCTTTACGTTTACAATTTTGTTTATTCAG
CTCTTTTACAATTGCTTTAACTTTTCCATTCTTATTTTTCAGATTGCTTTGATTTCCACGATCGTGAGCATTAAA
AGTTTAGGCGTGATACTGACCGGTTAAATTTCCCTTAATTGAAATTTAACTACATATTTAAACAGCAACATAGT
CCCTTTTGCCTAAAATGTCATCAAAAACTGGGAGGAAAAATATCATAGCAACAATCTGAATGAATTCGGAGAT
TCAGATCTGTTGTCACAAAAATTGTTGGATAAAATGTGCGAAAGTGTGTTTGTCAAATTGTTTAGCCAAAATT
ATTTGTTTACATAGTTGTTCAGCGTAGCACGAGGCTGTTTTCCTTAATTCTTAACAACATAGATACACTCGCC
TTGGCTAATTTACACCGGAACCTAAAGCCGATTAACAATAGGAAAATAGTCCTACGACAAATAGCCCGCCTT
TTGACATATGAACACATAACTAAACTACCACACAGCTCACCAAAAACATAAAATAAATTCGGAAAGTATAGCA
CCAAAGCTGAAATTTCAAGGAGATGCTGCTCAAATTTCATACTTGATGTCAAACAACGACGACCCCATAGCA
ACAACAACAACAACAGCAGAAAGAGAAGGAAAAGGAACAACTGGAATAACTGTAAGCTAGCGAACAGAGGG
AGAGAGGCAATCTCTGTACACACACCTGTCGTCCAGCGGGGGAAAGAGCAAAAGAGAGCGACGGATCTCC
CGTGTGTGTTGTTCGCATGCGCGACATGCGCGGACGCTAAGTGAGTGTGAGTGAGTGCGCGAATGCGTGT
*GAGTGTGGGCGGTGAGGATAATTCGCTCAATTCGCACGCATCCCACAGA**T**CCTGCAGATCCTCCAGTTTGT*
TCGTCAAACAGAGTCAGCATTTCGGATCAAACCAGTTGCCTGTCAGCAACATTAACAGCCTCAACGTTGGAA
AAAAAAAAACAAAAGCAGCAGCGGCAGTCGATAAAAAAATCATTATCAAAATTACGGAAAAGCGATCAAAATT
CAAGCGAGATTAGCTAGGCGAAATGCAGCGACGAGAACGGCAAGCAAGGTGAGTTATGATTGTGTAATTGT
ATATCGATTATCCCCCAAATTCATAAAGAGGTAATGGTTCTAAGACTCTTTCAAAAATTAAAAATTAATCCTAC
CATTTAAGTTGGCATTGCTTTTGGCTACAGTATTGAAACCCATTTATAGTTTTCTATTGTCAATCGAAAATCAA
GATAAGCGAATCTAGCCCACTGGCTTTATCCCTGCTTTTGAATCTGAATCTTTTGTACGCCCCTTAGCTATAC
CTTCCAACTCCAAGCGGTACTCAAATTTTGACTAGAGACCATAGGCTTGCGACTATTTATAATTTTGCATTGA
*T*AAGCCGTGCTAAAAAACCATTGCCACTATCTACGGAAGCGACGTCTGTTCTCATAAATGTTCCATATTCGGT
ACTACAGTACACTGTTATCAGGCGTAGAAGCGGTTGGATATTTTGATAGCGATAAGATTATGGCTGTGTCGG
GAAACGCGTGTTTGCAAGTTTTAAAACCTAACAGTTTTGATTTGGTTGGCTTAGTTAACCAATAGAACTAGCT
TTTCTTTCACTCATAAAACTTTTTAAAATATTAACGAATGCAAACTGCGAATTTTTAAAGAAAATGAGTCTGGT
TTAAAATAATTTTATTTCGTTTAACATAAATAAATTTCCATAAAGCTAATTCCTTTTAATTTCCTAGCGAATACT
GTACATTGTCAAAAGTTCAAGCAACAGATTTAGTTGTAGCTGCCTTTTTTGCCGAACGATTTTATTTTTAGCAA
AGAGATGTTTCTATTTAAACTTGTGTGTTTGATTTTATTTTATTTTCGCAAGTGTTTTTGTTGCTTTTGGCTTAT
GGCGTCTGCTTTTTCTCGCCTCTGGCACGCCAGCCAAACTAAGAAGAAGAGCCATGAGAAGGGGGTGAATG
AAATGGGTTTCCAATGCAATACATAATACCCACATACATACATATGTGGGTGCGTGATAGATAGCACCGGTC
TTTCGTGTAGGAGAGCGGGAAACGAAGTGCACACAACTCACGCACATTATAAATATTTTATAGGCATTTC

Hop AA Sequence:

aGTCCTaCGTACAAATaGCCCGCCTTTTGACATATGAACACATAACTAAACTACCACACAGCTCACCAAAAACA
TAAAATAAATTCGGaAAGTATAGCACCAAAGCTGaAATTTCAAGGAGATGCTGCTCAAATtTCATACTTGATGT
CAAACAACGACGACCCCATAGCAACAACAACAACAACAGCAGAAAGAGAAGGAAAAGGAACAACTGGAAT
AACTGTAAGCTAGCGAACAGAGGGAGAGAGGCAATCTCTGTACACACACCTGTCGTCCAGCGGGGGAAAGAG
CAAAAGAGAGCGACGGATCTCCCGTGTGTGTTGTTCGCATGCGCGACATGCGCGGACGCTAAGTGAGTGTGA
GTGAGTGCGCGAATGCGTGTGAGTGTGGGCGGTGAGGATAATTCGCTCAATTCGCACGCATCCCACAGATCCT
GCAGATCCTCCAGTTTGTTCGTCAAACAGAGTCAGCATTTCGGATCAAACCAGTTGCCTGTCAGCAACATTAA
CAGCCTCAACGTTGGAAAAAAAAAAAACAAAAGCAGCAgCGGCaGTCGATAAAAAAaTCTTTaTCAAAaTTACGG
AAAAGCGaTCAAAaTTCAaGCGAGaTTAgcTAGGCgAAaTGCAgCGACgAgAACGGCAAGCAAGGTGAGTTATGA
TTGTCTAATTGTATATCGATTATCCCCCAAATCCATAACGAGGTAATGGTTCTAAgACTCTTTCAAAAATTAAA
AATTAATCCTACCATTTAAGTTGGCaTTGCTTTTGGCTACaGTATTGAAACCCaTTTATAGTTTTCTaTTGTCA

- No sign of p-element. Precise excision

File S3

Seizure Threshold Data

Charlatan

Genotype	Threshold
elavgal4bss1/+;chnRNAi	5.6
elavgal4bss1/+;chnRNAi	3.1
elavgal4bss1/+;chnRNAi	3.1
elavgal4bss1/+;chnRNAi	2.8

Gish

Df ED10639

Genotype	Threshold	Control Genotype	Threshold
parabss1/+;;Ed10936/+	21	parabss1/+;;Tm3,Sb/+	5.4
parabss1/+;;Ed10936/+	21	parabss1/+;;Tm3,Sb/+	5
parabss1/+;;Ed10936/+	21	parabss1/+;;Tm3,Sb/+	5
parabss1/+;;Ed10936/+	8.2	parabss1/+;;Tm3,Sb/+	5
parabss1/+;;Ed10936/+	16	parabss1/+;;Tm3,Sb/+	3.6
parabss1/+;;Ed10936/+	10	parabss1/+;;Tm3,Sb/+	21
parabss1/+;;Ed10936/+	16	parabss1/+;;Tm3,Sb/+	21
parabss1/+;;Ed10936/+	16	parabss1/+;;Tm3,Sb/+	16
parabss1/+;;Ed10936/+	48	parabss1/+;;Tm3,Sb/+	21
parabss1/+;;Ed10936/+	21	parabss1/+;;Tm3,Sb/+	16
parabss1/+;;Ed10936/+	38	parabss1/+;;Tm3,Sb/+	7.5
parabss1/+;;Ed10936/+	30	parabss1/+;;Tm3,Sb/+	5.6
parabss1/+;;Ed10936/+	21	parabss1/+;;Tm3,Sb/+	6.9
parabss1/+;;Ed10936/+	21	parabss1/+;;Tm3,Sb/+	10
parabss1/+;;Ed10936/+	21	parabss1/+;;Tm3,Sb/+	5

Genotype	Threshold	Control Genotype	Threshold
w;;Ed10639/+	56	w;;Tm3,Sb/+	48
w;;Ed10639/+	56	w;;Tm3,Sb/+	48
w;;Ed10639/+	56	w;;Tm3,Sb/+	38
w;;Ed10639/+	56	w;;Tm3,Sb/+	66
w;;Ed10639/+	56	w;;Tm3,Sb/+	48
w;;Ed10639/+	42	w;;Tm3,Sb/+	38
w;;Ed10639/+	48	w;;Tm3,Sb/+	38
w;;Ed10639/+	48	w;;Tm3,Sb/+	38
w;;Ed10639/+	48	w;;Tm3,Sb/+	56
w;;Ed10639/+	88	w;;Tm3,Sb/+	21
w;;Ed10639/+	80	w;;Tm3,Sb/+	21
w;;Ed10639/+	56	w;;Tm3,Sb/+	52
w;;Ed10639/+	48	w;;Tm3,Sb/+	72
w;;Ed10639/+	100	w;;Tm3,Sb/+	61
w;;Ed10639/+	38	w;;Tm3,Sb/+	21

gish04895

Genotype	Threshold	Control Genotype	Threshold
parabss1/+;;gish04895/+	10	parabss1/+;;Tm6,Dr/+	16
parabss1/+;;gish04895/+	10	parabss1/+;;Tm6,Dr/+	10
parabss1/+;;gish04895/+	21	parabss1/+;;Tm6,Dr/+	21
parabss1/+;;gish04895/+	10	parabss1/+;;Tm6,Dr/+	6.5
parabss1/+;;gish04895/+	10	parabss1/+;;Tm6,Dr/+	6.5
parabss1/+;;gish04895/+	8.5	parabss1/+;;Tm6,Dr/+	21
parabss1/+;;gish04895/+	48	parabss1/+;;Tm6,Dr/+	7.5
parabss1/+;;gish04895/+	16	parabss1/+;;Tm6,Dr/+	8.5
parabss1/+;;gish04895/+	8.8	parabss1/+;;Tm6,Dr/+	7.5
parabss1/+;;gish04895/+	8.5	parabss1/+;;Tm6,Dr/+	7.5
parabss1/+;;gish04895/+	8.5	parabss1/+;;Tm6,Dr/+	5.6
parabss1/+;;gish04895/+	8.5	parabss1/+;;Tm6,Dr/+	8.2
parabss1/+;;gish04895/+	42	parabss1/+;;Tm6,Dr/+	8.2
parabss1/+;;gish04895/+	6.9	parabss1/+;;Tm6,Dr/+	8.2
parabss1/+;;gish04895/+	16	parabss1/+;;Tm6,Dr/+	4
parabss1/+;;gish04895/+	16	parabss1/+;;Tm6,Dr/+	8.2
parabss1/+;;gish04895/+	21	parabss1/+;;Tm6,Dr/+	21
parabss1/+;;gish04895/+	8.2	parabss1/+;;Tm6,Dr/+	6.2
parabss1/+;;gish04895/+	42	parabss1/+;;Tm6,Dr/+	8.2
parabss1/+;;gish04895/+	8.2	parabss1/+;;Tm6,Dr/+	5
parabss1/+;;gish04895/+	10	parabss1/+;;Tm6,Dr/+	21
parabss1/+;;gish04895/+	30	parabss1/+;;Tm6,Dr/+	8.2
parabss1/+;;gish04895/+	7.5	parabss1/+;;Tm6,Dr/+	7.5
parabss1/+;;gish04895/+	8.2	parabss1/+;;Tm6,Dr/+	5.4
parabss1/+;;gish04895/+	6.5	parabss1/+;;Tm6,Dr/+	7.5

Genotype	Threshold	Control Genotype	Threshold
w;;gish08495/+	30	w;;Tm6,Dr/+	72
w;;gish08495/+	80	w;;Tm6,Dr/+	80
w;;gish08495/+	66	w;;Tm6,Dr/+	38
w;;gish08495/+	56	w;;Tm6,Dr/+	30
w;;gish08495/+	72	w;;Tm6,Dr/+	48
w;;gish08495/+	95	w;;Tm6,Dr/+	38
w;;gish08495/+	72	w;;Tm6,Dr/+	21
w;;gish08495/+	66	w;;Tm6,Dr/+	38
w;;gish08495/+	80	w;;Tm6,Dr/+	38
w;;gish08495/+	66	w;;Tm6,Dr/+	48
w;;gish08495/+	100	w;;Tm6,Dr/+	30
w;;gish08495/+	48	w;;Tm6,Dr/+	21
w;;gish08495/+	56	w;;Tm6,Dr/+	38
w;;gish08495/+	16	w;;Tm6,Dr/+	66
w;;gish08495/+	48	w;;Tm6,Dr/+	80

Genotype	Threshold	Control Genotype	Threshold
elavbss1/+;;gish rnai/+	46.6	elavbss1/+;;tm6/+	8.09
elavbss1/+;;gish rnai/+	10.44	elavbss1/+;;tm6/+	5.8
elavbss1/+;;gish rnai/+	10.44	elavbss1/+;;tm6/+	6.95

elavbss1/+;;gish rnai/+	27.3	elavbss1/+;;tm6/+	8.09
elavbss1/+;;gish rnai/+	37.7	elavbss1/+;;tm6/+	9.2
elavbss1/+;;gish rnai/+	9.2	elavbss1/+;;tm6/+	8.09
elavbss1/+;;gish rnai/+	76.2	elavbss1/+;;tm6/+	9.2
elavbss1/+;;gish rnai/+	37.7	elavbss1/+;;tm6/+	8.09
elavbss1/+;;gish rnai/+	27.3	elavbss1/+;;tm6/+	9.2
elavbss1/+;;gish rnai/+	9.91	elavbss1/+;;tm6/+	9.2

Genotype	Threshold	Control Genotype	Threshold
eas;;gish04895/+	7.8	eas;;Tm6,Dr/+	5.85
eas;;gish04895/+	5.85	eas;;Tm6,Dr/+	3.41
eas;;gish04895/+	11.25	eas;;Tm6,Dr/+	8.77
eas;;gish04895/+	7.32	eas;;Tm6,Dr/+	5.85
eas;;gish04895/+	5.85	eas;;Tm6,Dr/+	7.8
eas;;gish04895/+	5.85	eas;;Tm6,Dr/+	3.41
eas;;gish04895/+	11.25	eas;;Tm6,Dr/+	5.85
eas;;gish04895/+	5.85	eas;;Tm6,Dr/+	5.85
eas;;gish04895/+	13	eas;;Tm6,Dr/+	5.85
eas;;gish04895/+	11.25	eas;;Tm6,Dr/+	5.22

File S4

Bang-Sensitivity Data

Charlatan

elavgal4c155;chnRNAi/+	0%
Df Exel7135/Cyo	0%
Recovery Time (s)	
parabss1;Df Exel7135/+	363
parabss1;+/Cyo	264
parabss1;Df BSC346/+	89.9
parabss1;Cyo/+	125.4
parabss1;Df BSC651/+	268
parabss1;Cyo/+	98.5
elavgal4c155parabss1;chnRNAi/+	261.9
elavgal4c155parabss1;+/+	105.6

Df Ed10639

parabss1/+;;Df Ed10639/+	10.6%
parabss1/+;;+/Tm3,Sb	82.1%

Df Exel7329

parabss1/+;;Df Exel7329/+	11.1%
parabss1/+;;+/Tm6B	87.4%

DfExel16269

parabss1/+;;Df Exel16269/+	95%
parabss1/+;;+/Tm6B	81%

Gish

parabss1/+;;gish04895/+	38.6%
parabss1/+;;Tm6, Dr	90.5%
parabss1/+;;gishKG03891/+	71.1%
parabss1/+;;Tm3,Sb	80.4%
parabss1/+;;gishDG16412/+	72.7%
parabss1/+;;Tm6B	87.7%
parabss1/+;;gishe01759/+	91.3%

parabss1/+;;Tm3,Sb	96%
parabss1/+;;gish04895excision	63.4%
parabss1/+;;Tm6B	61.95
elavgal4c155parabss1/+;;gishRNAi/+	14.0%
elavgal4c155parabss1/+;;+/Balancer	57.0%
parabss1;;Df Ed10639/+	100%
parabss1;;+/Tm3,Sb	100%
parabss1;;gish04895/+	100%
parabss1;;+/Tm6,Dr	100%
elavgal4c155parabss1;;gishRNAi/+	100%
elavgal4c155parabss1;;+/Balancer	100%
eas;;gish04895/+	100%
eas;;+/Tm6,Dr	100%
**Other**	
parabss1/+;;bor^{c05496}/+	78.5%
parabss1/+;;+/Tm6B	86.2%
parabss1/+;;tara1/+	87.1%
parabss1/+;;+/Tm3,Sb	78.9%
elavgal4c155parabss1;;arrRNAi/+	64.8%
elavgal4c155parabss1;;Tm6B/+	92.0%
The following are	
elavgal4c155parabss1;wgRNAi/+	30.3%
elavgal4c155parabss1;+/tft	52.2%
elavgal4c155parabss1;panRNAi/+	37.0%
elavgal4c155parabss1;+/tft	39.3%
