# Supplementary material for: Drosophila as a Model for Intractable Epilepsy: Gilgamesh Suppresses Seizures in parabss1 Heterozygote Flies
Source: G3 (Bethesda). 2013 Aug 1;3(8):1399–407. doi: 10.1534/g3.113.006130 (PMC3737179; doi:10.1534/g3.113.006130)
Supplement: Supporting Information [file supp_g3.113.006130_FileS1.pdf]

**File S1**

**Screen Data**

| Stock (Bloomington) | Chromosome | Df BS% | Bal BS% | Rec time Df | Rec time Bal | Difference |
|---|---|---|---|---|---|---|
| 7532 | 2 | 50 | 32 | 73 | 94 | 21 |
| 7533 | 2 | 96 | 68 | 155 | 152 | -3 |
| 7534 | 2 | 96 | 90 | 172 | 210 | 38 |
| 7535 | 2 | 96 | 84 | 114 | 120 | 6 |
| 7536 | 2 | 40 | 16 | 95 | 115 | 20 |
| 7537 | 2 | 86 | 55 | 97 | 127 | 30 |
| 7538 | 2 | 76 | 51 | 215 | 85 | -130 |
| 7539 | 2 | 92 | 70 | 160 | 177 | 17 |
| 7540 | 2 | 88 | 75 | 90 | 82 | -8 |
| 7541 | 2 | 89 | 90 | 171 | 191 | 20 |
| 7543 | 2 | 86 | 53 | 73 | 126 | 53 |
| 7544 | 2 | 91 | 75 | 132 | 129 | -3 |
| 7545 | 2 | 72 | 48 | 131 | 159 | 28 |
| 7546 | 2 | 98 | 71 | 205 | 190 | -15 |
| 7547 | 2 | 45 | 42 | 85 | 116 | 31 |
| 7548 | 2 | 71 | 59 | 90 | 106 | 16 |
| 7549 | 2 | 76 | 47 | 131 | 132 | 1 |
| 7551 | 2 | 89 | 82 | 143 | 126 | -17 |
| 7553 | 2 | 98 | 69 | 217 | 118 | -99 |
| 7554 | 2 | 54 | 18 | 128 | 108 | -20 |
| 7556 | 2 | 98 | 65 | 216 | 129 | -87 |
| 7557 | 2 | 94 | 85 | 186 | 216 | 30 |
| 7558 | 2 | 55 | 44 | 306 | 135 | -171 |
| 7559 | 2 | 76 | 85 | 284 | 215 | -69 |
| 7561 | 2 | 78 | 65 | 172 | 215 | 43 |
| 7748 | 2 | 81 | 64 | 97 | 138 | 41 |
| 7749 | 2 | 94 | 53 | 77 | 123 | 46 |
| 7750 | 2 | 2 | 72 | 96 | 121 | 25 |
| 7858 | 2 | 95 | 81 | 163 | 184 | 21 |

| | | | | | | |
|---|---|---|---|---|---|---|
| 7859 | 2 | 84 | 45 | 232 | 102 | -130 |
| 7860 | 2 | 93 | 57 | 144 | 128 | -16 |
| 7862 | 2 | 88 | 73 | 119 | 157 | 38 |
| 7863 | 2 | 97 | 70 | 143 | 221 | 78 |
| 7864 | 2 | 89 | 68 | 131 | 139 | 8 |
| 7867 | 2 | 92 | 72 | 103 | 105 | 2 |
| 7869 | 2 | 94 | 75 | 120 | 157 | 37 |
| 7870 | 2 | 92 | 34 | 146 | 204 | 58 |
| 7871 | 2 | 91 | 81 | 145 | 190 | 45 |
| 7872 | 2 | 82 | 30 | 198 | 136 | -62 |
| 7873 | 2 | 92 | 95 | 142 | 214 | 72 |
| 7875 | 2 | 94 | 50 | 162 | 194 | 32 |
| 7876 | 2 | 94 | 44 | 106 | 111 | 5 |
| 7877 | 2 | 95 | 66 | 118 | 156 | 38 |
| 7879 | 2 | 86 | 47 | 264 | 164 | -100 |
| 7880 | 2 | 91 | 81 | 161 | 174 | 13 |
| 7881 | 2 | 68 | 47 | 99 | 115 | 16 |
| 7882 | 2 | 63 | 16 | 99 | 99 | 0 |
| 7883 | 2 | 79 | 32 | 111 | 101 | -10 |
| 7886 | 2 | 76 | 41 | 152 | 193 | 41 |
| 7887 | 2 | 86 | 51 | 114 | 99 | -15 |
| 7888 | 2 | 93 | 45 | 204 | 149 | -55 |
| 7890 | 2 | 75 | 6 | 118 | 120 | 2 |
| 7891 | 2 | 35 | 33 | 73 | 123 | 50 |
| 7893 | 2 | 94 | 73 | 144 | 161 | 17 |
| 7894 | 2 | 84 | 64 | 98 | 142 | 44 |
| 7895 | 2 | 42 | 8 | 88 | 106 | 18 |
| 7898 | 2 | 62 | 79 | 127 | 124 | -3 |
| 7900 | 2 | 85 | 57 | 109 | 175 | 66 |
| 7901 | 2 | 98 | 83 | 203 | 160 | -43 |
| 7902 | 2 | 82 | 63 | 104 | 147 | 43 |
| 7903 | 2 | 95 | 30 | 139 | 147 | 8 |
| 7906 | 2 | 91 | 89 | 192 | 239 | 47 |
| 7908 | 2 | 70 | 20 | 239 | 133 | -106 |

| | | | | | | |
|---|---|---|---|---|---|---|
| 7909 | 2 | 95 | 89 | 241 | 217 | -24 |
| 7916 | 2 | 70 | 18 | 116 | 122 | 6 |
| 7998 | 2 | | | 107 | 113 | 6 |
| 9064 | 2 | 76 | 93 | | | 0 |
| 24931 | 2 | 48 | 98 | | | 0 |
| 8082 | 3 | 77 | 90 | 92 | 121 | 29 |
| 9481 | 3 | 11 | 82 | 76 | 75 | -1 |
| 9482 | 3 | 76 | 85 | | | 0 |
| 7736 | 3 | 95 | 81 | | | 0 |
| 7987 | 3 | 94 | 81 | | | 0 |
| 7984 | 3 | 89 | 87 | | | 0 |
| 7597 | 3 | 73 | 79 | | | 0 |
| 25689 | 3 | 70 | 79 | | | 0 |
| 24140 | 3 | 51 | 82 | 124 | 112 | -12 |
| 8962 | 3 | 95 | 98 | 115 | 88 | -27 |
| 24143 | 3 | 87 | 68 | 83 | 79 | -4 |
| 8065 | 3 | 82 | 69 | 116 | 102 | -14 |
| 7623 | 3 | 47 | 61 | 256 | 248 | -8 |
| 8047 | 3 | 53 | 99 | 140 | 136 | -4 |
| 8048 | 3 | 100 | 99 | 122 | 135 | 13 |
| 9693 | 3 | 94 | 91 | 221 | 229 | 8 |
| 8053 | 3 | 96 | 99 | 164 | 135 | -29 |
| 7562 | 3 | 99 | 99 | 108 | 120 | 12 |
| 8096 | 3 | 85 | 100 | 178 | 175 | -3 |
| 24410 | 3 | 96 | 100 | 169 | 162 | -7 |
| 7570 | 3 | 75 | 98 | 189 | 198 | 9 |
| 8059 | 3 | 71 | 96 | 159 | 160 | 1 |
| 7571 | 3 | 98 | 99 | | | 0 |
| 24415 | 3 | 96 | 100 | | | 0 |
| 9701 | 3 | 100 | 97 | 185 | 172 | -13 |
| 24941 | 3 | 89 | 100 | 115 | 113 | -2 |
| 8070 | 3 | 90 | 92 | 163 | 178 | 15 |
| 8069 | 3 | 65 | 95 | 74 | 67 | -7 |
| 23668 | 3 | 77 | 99 | 108 | 111 | 3 |

| | | | | | | |
|---|---|---|---|---|---|---|
| 8075 | 3 | 74 | 77 | 155 | 140 | -15 |
| 8072 | 3 | 50 | 100 | 110 | 107 | -3 |
| 8074 | 3 | 98 | 90 | 152 | 146 | -6 |
| 8100 | 3 | 79 | 94 | 150 | 127 | -23 |
| 8099 | 3 | 95 | 99 | 157 | 145 | -12 |
| 8081 | 3 | 95 | 95 | 200 | 213 | 13 |
| 8080 | 3 | 18 | 95 | 110 | 114 | 4 |
| 2492 | 3 | 76 | 92 | 116 | 129 | 13 |
| 8082 | 3 | 89 | 96 | 119 | 130 | 11 |
| 24952 | 3 | 98 | 98 | 184 | 173 | -11 |
| 8088 | 3 | 67 | 96 | 104 | 127 | 23 |
| 8101 | 3 | 85 | 83 | 145 | 113 | -32 |
| 9700 | 3 | 98 | 96 | 162 | 154 | -8 |
| 8967 | 3 | 82 | 96 | 101 | 82 | -19 |
| 7623 | 3 | 88 | 90 | 319 | 306 | -13 |
| 9629 | 3 | 100 | 100 | 223 | 226 | 3 |
| 24968 | 3 | 97 | 91 | 192 | 197 | 5 |
| 8682 | 3 | 83 | 83 | 96 | 77 | -19 |
| 24990 | 3 | 93 | 68 | 125 | 89 | -36 |
| 9080 | 3 | 93 | 77 | 74 | 80 | 6 |
| 9084 | 3 | 45 | 90 | 116 | 96 | -20 |
| 9090 | 3 | 53 | 69 | 107 | 143 | 36 |
| 25019 | 3 | 97 | 99 | 150 | 162 | 12 |
| 24137 | 3 | 60 | 83 | 103 | 78 | -25 |
| 6962 | 3 | 44 | 59 | 107 | 109 | 2 |
| 7983 | 3 | 89 | 95 | 190 | 175 | -15 |
| 7737 | 3 | 96 | 95 | 109 | 116 | 7 |
| 9207 | 3 | 55 | 92 | 95 | 103 | 8 |
| 8104 | 3 | 53 | 85 | 114 | 88 | -26 |
| 25014 | 3 | 78 | 91 | 119 | 132 | 13 |
| 9486 | 3 | 100 | 99 | 170 | 161 | -9 |
| 24139 | 3 | 43 | 67 | 119 | 79 | -40 |
| 8962 | 3 | 37 | 65 | 73 | 56 | -17 |
| 24915 | 3 | 90 | 93 | | | 0 |

| | | | | | | |
|---|---|---|---|---|---|---|
| 25001 | 3 | 96 | 83 | 123 | 144 | 21 |
| 8097 | 3 | 6 | 81 | 117 | 99 | -18 |
| 7562 | 3 | 99 | 95 | 149 | 160 | 11 |
| 8047 | 3 | 22 | 77 | 126 | 88 | -38 |
| 8048 | 3 | 90 | 93 | 107 | 82 | -25 |
| 9693 | 3 | 97 | 94 | 154 | 148 | -6 |
| 7570 | 3 | 86 | 93 | 91 | 98 | 7 |
| 8096 | 3 | 50 | 89 | 89 | 76 | -13 |
| 8073 | 3 | 83 | 92 | 106 | 61 | -45 |
| 24410 | 3 | 89 | 84 | 146 | 97 | -49 |
| 7571 | 3 | 91 | 100 | 182 | 99 | -83 |
| 24392 | 3 | 94 | 92 | 170 | 87 | -83 |
| 8060 | 3 | 69 | 84 | 32 | 78 | 46 |
| 24941 | 3 | 78 | 89 | 152 | 116 | -36 |
| 24915 | 3 | 86 | 95 | 160 | 102 | -58 |
| 8061 | 3 | 80 | 50 | 101 | 59 | -42 |
| 9701 | 3 | 97 | 99 | 149 | 100 | -49 |
| 24413 | 3 | 99 | 98 | 190 | 134 | -56 |
| 8065 | 3 | 77 | 88 | 137 | 90 | -47 |
| 8974 | 3 | 94 | 94 | 170 | 98 | -72 |
| 7929 | 3 | 94 | 94 | 156 | 116 | -40 |
| 24415 | 3 | 81 | 82 | 124 | 91 | -33 |
| 23668 | 3 | 80 | 89 | 107 | 94 | -13 |
| 9355 | 3 | 70 | 82 | 92 | 81 | -11 |
| 8066 | 3 | 76 | 87 | 120 | 95 | -25 |
| 8068 | 3 | 72 | 83 | 56 | 75 | 19 |
| 8070 | 3 | 84 | 90 | 79 | 64 | -15 |
| 8073 | 3 | 91 | 96 | 99 | 58 | -41 |
| 8097 | 3 | 37 | 88 | 136 | 105 | -31 |
| 8074 | 3 | 76 | 91 | 128 | 97 | -31 |
