# Supplementary material for: Drosophila as a Model for Intractable Epilepsy: Gilgamesh Suppresses Seizures in parabss1 Heterozygote Flies
Source: G3 (Bethesda). 2013 Aug 1;3(8):1399–407. doi: 10.1534/g3.113.006130 (PMC3737179; doi:10.1534/g3.113.006130)
Supplement: Supporting Information [file supp_g3.113.006130_FileS2.pdf]

File S2

Sequence Data

Red C is insertion point of P-element from Bloomington stock 11790. Blue letters are where primers were designed to verify presence of insert.

4/8/10 - Primers designed to be used with P-element inverse repeat primer. If insert is there, product should be seen following PCR.

- Gish Forward Primer – gish11790_f (to be used with Repeat Reverse Primer) - CGT AGC ACG AGG CTG TTT TC (expected size ~516)
- Gish Reverse Primer – gish11790_r (to be used with Repeat Forward Primer) - CGC AAG CCT ATG GTC TCT AG (expected size ~523)

Gilgamesh gene region sequence near insertion (from Flybase)

ATATGGCCAAAAGTCGAGTAGTTACACCCTCTCTGCCTCTTTGCCCCTCTGCCTTTCTTGGTTTTCTCTCTCT
TATTCTCTTTGGGTCTTTGGCCATCGCCGCCTTGGCTCGTTTTTTGCATCGAATAACAAAACTTGAAAATTTA
TAAAAATAAACGAAATCACGTACGGAAATATTTACTTCGACCTTGCGGCCCCAGAGATTTTTCCAACAATTTA
TACGGCGCGGTGGCCATGGCAGCTAATTAGCCTAATTTTCTGTAATTTTCTAAATTTTAATTTGGTACCTTTTA
GAAGAGAGTAACTTTCATTTGTGCGACGATGACTTTAAAAGTTTTAAGTTAAAGTCAATAGTAACTCTTACTAA
ATTAAATGAAAATCAATTGAAAAAAAAAAAACAAATTTAGTTAGCGCTCTCATCAGTGCTTTTAGTGTGACCAG
CATACGCTATGGCTGGCCTAACATACCAAAAGAAATACCGCAGTTTCGTACATTCATTAGTCTATTTACTATT
TTTATTATCCATACAGAATGAAAAATGTTTAAATCGTCGGTAATATGAGTTTTTTTATATTTTGTATCAGTTAAC
ATGATCTTTCGTCAAAACATTCAAAGCGCTTGGAATTAAGACCGATCTTTACGTTTACAATTTTGTTTATTCAG
CTCTTTTACAATTGCTTTAACTTTTCCATTCTTATTTTTCAGATTGCTTTGATTTCCACGATCGTGAGCATTAAA
AGTTTAGGCGTGATACTGACCGGTTAAATTTCCCTTAATTGAAATTTAACTACATATTTAAACAGCAACATAGT
CCCTTTTGCCTAAAATGTCATCAAAAACTGGGAGGAAAAATATCATAGCAACAATCTGAATGAATTCGGAGAT
TCAGATCTGTTGTCACAAAAATTGTTGGATAAAATGTGCGAAAGTGTGTTTGTCAAATTGTTTAGCCAAAATT
ATTTGTTTACATAGTTGTTCAGCGTAGCACGAGGCTGTTTTCCTTAATTCTTAACAACATAGATACACTCGCC
TTGGCTAATTTACACCGGAACCTAAAGCCGATTAACAATAGGAAAATAGTCCTACGACAAATAGCCCGCCTT
TTGACATATGAACACATAACTAAACTACCACACAGCTCACCAAAAACATAAAATAAATTCGGAAAGTATAGCA
CCAAAGCTGAAATTTCAAGGAGATGCTGCTCAAATTTCATACTTGATGTCAAACAACGACGACCCCATAGCA
ACAACAACAACAACAGCAGAAAGAGAAGGAAAAGGAACAACTGGAATAACTGTAAGCTAGCGAACAGAGGG
AGAGAGGCAATCTCTGTACACACACCTGTCGTCCAGCGGGGGAAAGAGCAAAAGAGAGCGACGGATCTCC
CGTGTGTGTTGTTCGCATGCGCGACATGCGCGGACGCTAAGTGAGTGTGAGTGAGTGCGCGAATGCGTGT
GAGTGTGGGCGGTGAGGATAATTCGCTCAATTCGCACGCATCCCACAGATCCTGCAGATCCTCCAGTTTGT
TCGTCAAACAGAGTCAGCATTTCGGATCAAACCAGTTGCCTGTCAGCAACATTAACAGCCTCAACGTTGGAA
AAAAAAAAACAAAAGCAGCAGCGGCAGTCGATAAAAAAATCATTATCAAAATTACGGAAAAGCGATCAAAATT
CAAGCGAGATTAGCTAGGCGAAATGCAGCGACGAGAACGGCAAGCAAGGTGAGTTATGATTGTGTAATTGT
ATATCGATTATCCCCCAAATTCATAAAGAGGTAATGGTTCTAAGACTCTTTCAAAAATTAAAAATTAATCCTAC
CATTTAAGTTGGCATTGCTTTTGGCTACAGTATTGAAACCCATTTATAGTTTTCTATTGTCAATCGAAAATCAA
GATAAGCGAATCTAGCCCACTGGCTTTATCCCTGCTTTTGAATCTGAATCTTTTGTACGCCCCTTAGCTATAC
CTTCCAACTCCAAGCGGTACTCAAATTTTGACTAGAGACCATAGGCTTGCGACTATTTATAATTTTGCATTGA
*T*AAGCCGTGCTAAAAAACCATTGCCACTATCTACGGAAGCGACGTCTGTTCTCATAAATGTTCCATATTCGGT
ACTACAGTACACTGTTATCAGGCGTAGAAGCGGTTGGATATTTTGATAGCGATAAGATTATGGCTGTGTCGG
GAAACGCGTGTTTGCAAGTTTTAAAACCTAACAGTTTTGATTTGGTTGGCTTAGTTAACCAATAGAACTAGCT
TTTCTTTCACTCATAAAACTTTTTAAAATATTAACGAATGCAAACTGCGAATTTTTAAAGAAAATGAGTCTGGT
TTAAAATAATTTTATTTCGTTTAACATAAATAAATTTCCATAAAGCTAATTCCTTTTAATTTCCTAGCGAATACT
GTACATTGTCAAAAGTTCAAGCAACAGATTTAGTTGTAGCTGCCTTTTTTGCCGAACGATTTTATTTTTAGCAA
AGAGATGTTTCTATTTAAACTTGTGTGTTTGATTTTATTTTATTTTCGCAAGTGTTTTTGTTGCTTTTGGCTTAT
GGCGTCTGCTTTTTCTCGCCTCTGGCACGCCAGCCAAACTAAGAAGAAGAGCCATGAGAAGGGGGTGAATG
AAATGGGTTTCCAATGCAATACATAATACCCACATACATACATATGTGGGTGCGTGATAGATAGCACCGGTC
TTTCGTGTAGGAGAGCGGGAAACGAAGTGCACACAACTCACGCACATTATAAATATTTTATAGGCATTTC

Hop AA Sequence:

aGTCCTaCGTACAAATaGCCCGCCTTTTGACATATGAACACATAACTAAACTACCACACAGCTCACCAAAAACA
TAAAATAAATTCGGaAAGTATAGCACCAAAGCTGaAATTTCAAGGAGATGCTGCTCAAATtTCATACTTGATGT
CAAACAACGACGACCCCATAGCAACAACAACAACAACAGCAGAAAGAGAAGGAAAAGGAACAACTGGAAT
AACTGTAAGCTAGCGAACAGAGGGAGAGAGGCAATCTCTGTACACACACCTGTCGTCCAGCGGGGGAAAGAG
CAAAAGAGAGCGACGGATCTCCCGTGTGTGTTGTTCGCATGCGCGACATGCGCGGACGCTAAGTGAGTGTGA
GTGAGTGCGCGAATGCGTGTGAGTGTGGGCGGTGAGGATAATTCGCTCAATTCGCACGCATCCCACAGATCCT
GCAGATCCTCCAGTTTGTTCGTCAAACAGAGTCAGCATTTCGGATCAAACCAGTTGCCTGTCAGCAACATTAA
CAGCCTCAACGTTGGAAAAAAAAAAAACAAAAGCAGCAgCGGCaGTCGATAAAAAAaTCTTTaTCAAAaTTACGG
AAAAGCGaTCAAAaTTCAaGCGAGaTTAgcTAGGCgAAaTGCAgCGACgAgAACGGCAAGCAAGGTGAGTTATGA
TTGTCTAATTGTATATCGATTATCCCCCAAATCCATAACGAGGTAATGGTTCTAAgACTCTTTCAAAAATTAAA
AATTAATCCTACCATTTAAGTTGGCaTTGCTTTTGGCTACaGTATTGAAACCCaTTTATAGTTTTCTaTTGTCA

- No sign of p-element. Precise excision
