# Supplementary material for: Drosophila as a Model for Intractable Epilepsy: Gilgamesh Suppresses Seizures in parabss1 Heterozygote Flies
Source: G3 (Bethesda). 2013 Aug 1;3(8):1399–407. doi: 10.1534/g3.113.006130 (PMC3737179; doi:10.1534/g3.113.006130)
Supplement: Supporting Information [file supp_g3.113.006130_FileS3.pdf]

File S3

Seizure Threshold Data

Charlatan

Genotype	Threshold
elavgal4bss1/+;chnRNAi	5.6
elavgal4bss1/+;chnRNAi	3.1
elavgal4bss1/+;chnRNAi	3.1
elavgal4bss1/+;chnRNAi	2.8

Gish

Df ED10639

Genotype	Threshold	Control Genotype	Threshold
parabss1/+;;Ed10936/+	21	parabss1/+;;Tm3,Sb/+	5.4
parabss1/+;;Ed10936/+	21	parabss1/+;;Tm3,Sb/+	5
parabss1/+;;Ed10936/+	21	parabss1/+;;Tm3,Sb/+	5
parabss1/+;;Ed10936/+	8.2	parabss1/+;;Tm3,Sb/+	5
parabss1/+;;Ed10936/+	16	parabss1/+;;Tm3,Sb/+	3.6
parabss1/+;;Ed10936/+	10	parabss1/+;;Tm3,Sb/+	21
parabss1/+;;Ed10936/+	16	parabss1/+;;Tm3,Sb/+	21
parabss1/+;;Ed10936/+	16	parabss1/+;;Tm3,Sb/+	16
parabss1/+;;Ed10936/+	48	parabss1/+;;Tm3,Sb/+	21
parabss1/+;;Ed10936/+	21	parabss1/+;;Tm3,Sb/+	16
parabss1/+;;Ed10936/+	38	parabss1/+;;Tm3,Sb/+	7.5
parabss1/+;;Ed10936/+	30	parabss1/+;;Tm3,Sb/+	5.6
parabss1/+;;Ed10936/+	21	parabss1/+;;Tm3,Sb/+	6.9
parabss1/+;;Ed10936/+	21	parabss1/+;;Tm3,Sb/+	10
parabss1/+;;Ed10936/+	21	parabss1/+;;Tm3,Sb/+	5

Genotype	Threshold	Control Genotype	Threshold
w;;Ed10639/+	56	w;;Tm3,Sb/+	48
w;;Ed10639/+	56	w;;Tm3,Sb/+	48
w;;Ed10639/+	56	w;;Tm3,Sb/+	38
w;;Ed10639/+	56	w;;Tm3,Sb/+	66
w;;Ed10639/+	56	w;;Tm3,Sb/+	48
w;;Ed10639/+	42	w;;Tm3,Sb/+	38
w;;Ed10639/+	48	w;;Tm3,Sb/+	38
w;;Ed10639/+	48	w;;Tm3,Sb/+	38
w;;Ed10639/+	48	w;;Tm3,Sb/+	56
w;;Ed10639/+	88	w;;Tm3,Sb/+	21
w;;Ed10639/+	80	w;;Tm3,Sb/+	21
w;;Ed10639/+	56	w;;Tm3,Sb/+	52
w;;Ed10639/+	48	w;;Tm3,Sb/+	72
w;;Ed10639/+	100	w;;Tm3,Sb/+	61
w;;Ed10639/+	38	w;;Tm3,Sb/+	21

gish04895

Genotype	Threshold	Control Genotype	Threshold
parabss1/+;;gish04895/+	10	parabss1/+;;Tm6,Dr/+	16
parabss1/+;;gish04895/+	10	parabss1/+;;Tm6,Dr/+	10
parabss1/+;;gish04895/+	21	parabss1/+;;Tm6,Dr/+	21
parabss1/+;;gish04895/+	10	parabss1/+;;Tm6,Dr/+	6.5
parabss1/+;;gish04895/+	10	parabss1/+;;Tm6,Dr/+	6.5
parabss1/+;;gish04895/+	8.5	parabss1/+;;Tm6,Dr/+	21
parabss1/+;;gish04895/+	48	parabss1/+;;Tm6,Dr/+	7.5
parabss1/+;;gish04895/+	16	parabss1/+;;Tm6,Dr/+	8.5
parabss1/+;;gish04895/+	8.8	parabss1/+;;Tm6,Dr/+	7.5
parabss1/+;;gish04895/+	8.5	parabss1/+;;Tm6,Dr/+	7.5
parabss1/+;;gish04895/+	8.5	parabss1/+;;Tm6,Dr/+	5.6
parabss1/+;;gish04895/+	8.5	parabss1/+;;Tm6,Dr/+	8.2
parabss1/+;;gish04895/+	42	parabss1/+;;Tm6,Dr/+	8.2
parabss1/+;;gish04895/+	6.9	parabss1/+;;Tm6,Dr/+	8.2
parabss1/+;;gish04895/+	16	parabss1/+;;Tm6,Dr/+	4
parabss1/+;;gish04895/+	16	parabss1/+;;Tm6,Dr/+	8.2
parabss1/+;;gish04895/+	21	parabss1/+;;Tm6,Dr/+	21
parabss1/+;;gish04895/+	8.2	parabss1/+;;Tm6,Dr/+	6.2
parabss1/+;;gish04895/+	42	parabss1/+;;Tm6,Dr/+	8.2
parabss1/+;;gish04895/+	8.2	parabss1/+;;Tm6,Dr/+	5
parabss1/+;;gish04895/+	10	parabss1/+;;Tm6,Dr/+	21
parabss1/+;;gish04895/+	30	parabss1/+;;Tm6,Dr/+	8.2
parabss1/+;;gish04895/+	7.5	parabss1/+;;Tm6,Dr/+	7.5
parabss1/+;;gish04895/+	8.2	parabss1/+;;Tm6,Dr/+	5.4
parabss1/+;;gish04895/+	6.5	parabss1/+;;Tm6,Dr/+	7.5

Genotype	Threshold	Control Genotype	Threshold
w;;gish08495/+	30	w;;Tm6,Dr/+	72
w;;gish08495/+	80	w;;Tm6,Dr/+	80
w;;gish08495/+	66	w;;Tm6,Dr/+	38
w;;gish08495/+	56	w;;Tm6,Dr/+	30
w;;gish08495/+	72	w;;Tm6,Dr/+	48
w;;gish08495/+	95	w;;Tm6,Dr/+	38
w;;gish08495/+	72	w;;Tm6,Dr/+	21
w;;gish08495/+	66	w;;Tm6,Dr/+	38
w;;gish08495/+	80	w;;Tm6,Dr/+	38
w;;gish08495/+	66	w;;Tm6,Dr/+	48
w;;gish08495/+	100	w;;Tm6,Dr/+	30
w;;gish08495/+	48	w;;Tm6,Dr/+	21
w;;gish08495/+	56	w;;Tm6,Dr/+	38
w;;gish08495/+	16	w;;Tm6,Dr/+	66
w;;gish08495/+	48	w;;Tm6,Dr/+	80

Genotype	Threshold	Control Genotype	Threshold
elavbss1/+;;gish rnai/+	46.6	elavbss1/+;;tm6/+	8.09
elavbss1/+;;gish rnai/+	10.44	elavbss1/+;;tm6/+	5.8
elavbss1/+;;gish rnai/+	10.44	elavbss1/+;;tm6/+	6.95

elavbss1/+;;gish rnai/+	27.3	elavbss1/+;;tm6/+	8.09
elavbss1/+;;gish rnai/+	37.7	elavbss1/+;;tm6/+	9.2
elavbss1/+;;gish rnai/+	9.2	elavbss1/+;;tm6/+	8.09
elavbss1/+;;gish rnai/+	76.2	elavbss1/+;;tm6/+	9.2
elavbss1/+;;gish rnai/+	37.7	elavbss1/+;;tm6/+	8.09
elavbss1/+;;gish rnai/+	27.3	elavbss1/+;;tm6/+	9.2
elavbss1/+;;gish rnai/+	9.91	elavbss1/+;;tm6/+	9.2

Genotype	Threshold	Control Genotype	Threshold
eas;;gish04895/+	7.8	eas;;Tm6,Dr/+	5.85
eas;;gish04895/+	5.85	eas;;Tm6,Dr/+	3.41
eas;;gish04895/+	11.25	eas;;Tm6,Dr/+	8.77
eas;;gish04895/+	7.32	eas;;Tm6,Dr/+	5.85
eas;;gish04895/+	5.85	eas;;Tm6,Dr/+	7.8
eas;;gish04895/+	5.85	eas;;Tm6,Dr/+	3.41
eas;;gish04895/+	11.25	eas;;Tm6,Dr/+	5.85
eas;;gish04895/+	5.85	eas;;Tm6,Dr/+	5.85
eas;;gish04895/+	13	eas;;Tm6,Dr/+	5.85
eas;;gish04895/+	11.25	eas;;Tm6,Dr/+	5.22
