# Supplementary material for: Drosophila as a Model for Intractable Epilepsy: Gilgamesh Suppresses Seizures in parabss1 Heterozygote Flies
Source: G3 (Bethesda). 2013 Aug 1;3(8):1399–407. doi: 10.1534/g3.113.006130 (PMC3737179; doi:10.1534/g3.113.006130)
Supplement: Supporting Information [file supp_g3.113.006130_FileS4.pdf]

File S4

Bang-Sensitivity Data

**Charlatan**	
elavgal4c155;chnRNAi/+	0%
Df Exel7135/Cyo	0%
Recovery Time (s)	
parabss1;Df Exel7135/+	363
parabss1;+/Cyo	264
parabss1;Df BSC346/+	89.9
parabss1;Cyo/+	125.4
parabss1;Df BSC651/+	268
parabss1;Cyo/+	98.5
elavgal4c155parabss1;chnRNAi/+	261.9
elavgal4c155parabss1;+/+	105.6
**Df Ed10639**	
parabss1/+;;Df Ed10639/+	10.6%
parabss1/+;;+/Tm3,Sb	82.1%
**Df Exel7329**	
parabss1/+;;Df Exel7329/+	11.1%
parabss1/+;;+/Tm6B	87.4%
**DfExel16269**	
parabss1/+;;Df Exel16269/+	95%
parabss1/+;;+/Tm6B	81%
**Gish**	
parabss1/+;;gish04895/+	38.6%
parabss1/+;;Tm6, Dr	90.5%
parabss1/+;;gishKG03891/+	71.1%
parabss1/+;;Tm3,Sb	80.4%
parabss1/+;;gishDG16412/+	72.7%
parabss1/+;;Tm6B	87.7%
parabss1/+;;gishe01759/+	91.3%

parabss1/+;;Tm3,Sb	96%
parabss1/+;;gish04895excision	63.4%
parabss1/+;;Tm6B	61.95
elavgal4c155parabss1/+;;gishRNAi/+	14.0%
elavgal4c155parabss1/+;;+/Balancer	57.0%
parabss1;;Df Ed10639/+	100%
parabss1;;+/Tm3,Sb	100%
parabss1;;gish04895/+	100%
parabss1;;+/Tm6,Dr	100%
elavgal4c155parabss1;;gishRNAi/+	100%
elavgal4c155parabss1;;+/Balancer	100%
eas;;gish04895/+	100%
eas;;+/Tm6,Dr	100%
**Other**	
parabss1/+;;bor^{c05496}/+	78.5%
parabss1/+;;+/Tm6B	86.2%
parabss1/+;;tara1/+	87.1%
parabss1/+;;+/Tm3,Sb	78.9%
elavgal4c155parabss1;;arrRNAi/+	64.8%
elavgal4c155parabss1;;Tm6B/+	92.0%
The following are	
elavgal4c155parabss1;wgRNAi/+	30.3%
elavgal4c155parabss1;+/tft	52.2%
elavgal4c155parabss1;panRNAi/+	37.0%
elavgal4c155parabss1;+/tft	39.3%
